# *Rhus coriaria* L. in tradition and innovation like natural dye

**DOI:** 10.1038/s41598-024-62528-8

**Published:** 2024-05-27

**Authors:** Pierpaolo Scarano, Antonello Prigioniero, Maria Tartaglia, Daniela Zuzolo, Maria Maisto, Maria Antonietta Ranauda, Rosario Schicchi, Anna Geraci, Rosaria Sciarrillo, Carmine Guarino

**Affiliations:** 1 Department of Science and Technology, University of Sannio, Via F. de Sanctis, Snc, 82100 Benevento, Italy; 2 Department of Agricultural, Food and Forestry Sciences, University of Palermo, Viale Delle Scienze, Ed. 4, 90128 Palermo, Italy; 3 Department of Biological, Chemical and Pharmaceutical Sciences and Technologies (STEBICEF), University of Palermo, Viale Delle Scienze, Ed. 16, 90128 Palermo, Italy

**Keywords:** Spice, Natural dye, Anthocyanin, Gallotannin, CIELAB, Textile, Natural products, Mass spectrometry, Sustainability

## Abstract

Nowadays, secondary raw materials (SRM) obtained from plant matrices are of great interest for circular economy, suitable for sustainable measures to reduce environmental impact. This work focused on the extraction, characterization and quantification of compounds obtained from leaves and fruits of the Sicilian sumac, *Rhus coriaria* L. and their application as natural dyes on textile fibres. Extractions were performed with Extractor Naviglio®, maceration and ultrasound assisted methods and food-grade solvents (aqueous and hydroalcoholic) to evaluate the yields for dye compounds. The presence of colouring molecules was evaluated by UV–Vis spectrophotometer, and the extracts selected for colouring were quantified and characterized by LC–MS. The results showed that Extractor Naviglio® achieved the best extraction yield, and the ethanol–water mixture extracts had a higher amount of total phenolic compounds (TPC) and a higher content of total colouring compounds (TCC). These extracts were selected for subsequent applications as dyes for linen, cotton and wool. The chemical profile of selected extracts was rich in compounds such as gallotannin and anthocyanin class. Fibre dyeing was verified by recording CIELAB colouring coordinates. The results suggest that the dyes obtained from *R. coriaria* can be of great interest for artisanal and industrial processes, in accordance with environmental sustainability.

## Introduction

International agreements on environmental safeguard are becoming increasingly stringent. Sectors such as food and textiles are undergoing profound changes in the use and marketing of synthetic dyes and auxiliary chemical compounds; this trend significantly promotes the use and application of natural dyes^1^, both in food and textile processes. In fact, natural dyes are less impactful on the environment and do not adversely affect health^2^. Such application is closely linked to the ethnobotanical tradition about dyeing plants^3^.

With a view to sustainability, the possibility to recover bioactive and technologically exploitable compounds to be used industrially with high-performance and eco-friendly techniques, can become key to reducing operating costs and offering new revenue strategies within the production chain^4,5^.

The goals of Agenda 2030^6^, promoting the principles of the circular economy, provide for the improvement of the sustainability in production processes and products themselves^7–9^. Returning to the use of natural compounds enhances endemic plants while pursuing the final product without negatively affecting the Sustainable Development Goals (SDGs) defined by the United Nations Organisation^6^.

*Rhus coriaria* L., belonging to the Anacardiaceae family, is commonly known as sumac (derived from 'sumaga', which means red in Syriac)^10^. This plant, generally, has both nutritional values and medicinal applications and is consumed as a spice or medicinal herb in Persia, Mediterranean countries and the Middle East^11–22^.

*R. coriaria* is an evergreen shrub, which can reach heights of 1 to 10 m, with hirsute young branches. Several parts of sumac (leaves, bark and flowers) contain functional compounds used for tinctures in the local ethnobotanical tradition^23^. They are rich in gallic acid, (bi)flavonoid, sugars and essential oils^16^. Studies have focused on their tannin and flavonoid content^24^, as they play a protective role against herbivores and pests; they also seem to influence the regulation of plant growth^4,25^. The abundance of tannins is an important characteristic of the genus *Rhus*. Tannins can be divided into two subclasses: hydrolysable and condensed, and *R. coriaria* is a great source of hydrolysable ones.

From a technological and industrial point of view, sumac contains colouring substances, which have been found to be tannins and anthocyanins^26^. In Turkish tradition, sumac leaves and twigs are used to obtain a black dye^27^, while its bark extract is used to produce yellow dye, used for wool and cotton^28^. In addition, thanks to the high fixing property, and excellent resistance to the attack of fungi and moulds, it was used for the treatment of wood to prevent natural decomposition phenomena^29^. One of the key reasons that could lead to a return of the use of these substances is that current technologies can overcome the production difficulties that once existed^30,31^.

Considering the potential of sumac for colouring applications, we evaluated the dyeing efficiency of extracts obtained from *R. coriaria* leaves and fruit, on animal (wool) and plant (linen and cotton) textiles. Firstly, the extracts were obtained by three different applications: maceration (ME), Extractor Naviglio® (NE)^32^ and ultrasound-assisted (UAE). Then, the extracts were quantified and characterisedby means of UV–Vis and LC–MS/MS, to define the best performing extraction technology. The extracts were used for textiles staining by three different mordating methods: without mordant, with lemon juice or rock alum mordants. The staining tests were evaluated by CIELAB data (CIELAB units expressing colour coordinate values). The application, efficiency and persistence of the dyeing on the fibres were evaluated on a point scale.

## Results

**Figure 1:**
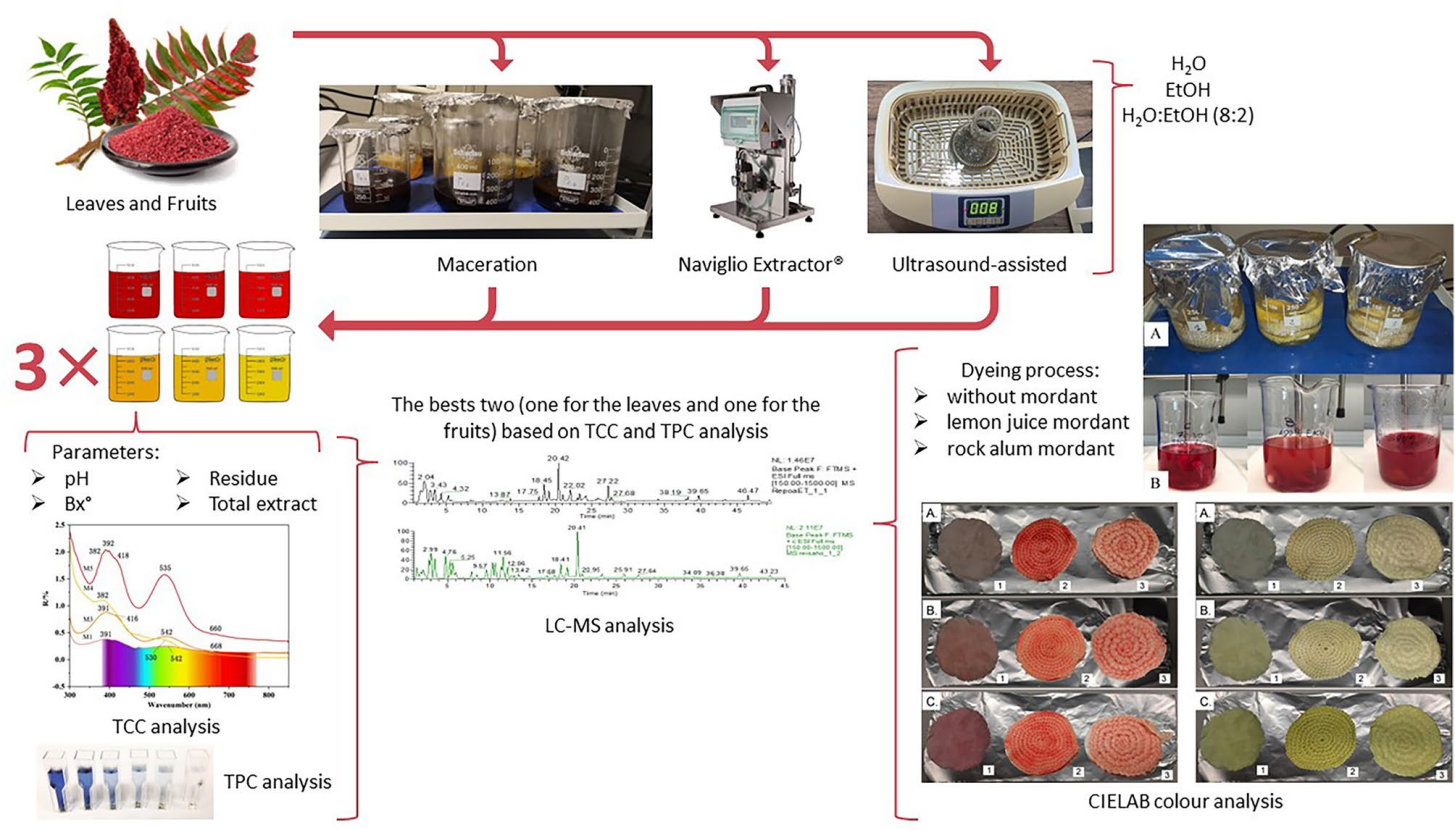
General workflow outline.

A flowchart of colouring experiment (extraction, analysis and colourant applications) is represented in Fig. 1.

### Parameters of the extracts

The performance of all the extraction techniques and solvents used were evaluated: the dry extracts obtained were quantified by weighing to evaluate the extraction yield of each method/solvent. Effective exhaustion was verified for each extracted plant sample. The values are reported in Supplementary Table 1.

Overall, relevant differences among the extraction techniques were evidenced (Supplementary Table 1) for all dry residues and total extract. Also, the same technique performed with different solvents led to extracts with different characteristics. No significant differences were observed for °Bx and pH of the extracts.

The total phenolic compounds (TPC) of the different *R. coriaria* extracts were estimated with the Folin-Ciocȃlteu reagent method^33^ and reported in Supplementary Table 2.

In particular, for both the fruits and leaves, the concentration in TPC in the EtOH:H_2_O mixture in a ratio of 8:2 (v/v) (from now on E8) was higher than other solvents for all extraction methods (Supplementary Table 2); only in two cases, both in the leaf extract, the EtOH (from now on E1) was slightly higher (8.1 × 10^−2^ mol·L^−1^ of GAE versus 7.0 × 10^−2^ mol﻿·L^−1^ of GAE using ME, 13.4 × 10^−2^ mol﻿·L^−1^ of GAE versus 12.4 × 10^−2^ mol﻿·L^−1^ of GAE using UAE). This result demonstrated that the E8 is the most suitable solvent for the extraction of phenolic compounds. As regards the extraction method, the TPCs of both the fruit and the leaf are extracted in greater quantities, regardless of the type of solvent used, with NE compared to ME and UAE, considering the comparable extraction time (1 h for all extractions) and the ratio quantity of matrix/quantity of solvent used remained constant in all techniques (1:10); only in one case, UAE (3.9 × 10^−2^ mol﻿·L^−1^ of GAE) had a better result than ME and NE (1.1 × 10^−2^ mol﻿·L^−1^ of GAE and 3.7 × 10^−2^ mol﻿·L^-1^ of GAE, respectively), with H_2_O (from now on H1) as extraction solvent. The best results, therefore, were obtained when using NE and the E8 (0.12490 mol﻿·L^−1^ in fruit and 0.19297 mol﻿·L^−1^ of GAE).

### UV–Vis characterization of extracted dyes

The quantitative determination of the dye was carried out considering the maximum absorbance signal data, taken at the wavelength at the maximum absorption band of the dye compounds analysed. These data are reported in moles per liter referring to standard (mol﻿·L^−1^). The estimated amount of total colouring compounds (TCC) was in a range of 8.3–13.6 × 10^−3^ mol·L^−1^ (fruit) and 1.2–20.2 × 10^−3^ mol﻿·L^−1^ (leaf) (Table 1). The R^2^ range was 0.9817 to 0.9990, and the relative standard deviation (RSD) slope values among the measured samples were less than 5%.

**Table 1:** Total colouring compound content (TCC) of *R. coriaria* extracts.

	ME			NE			UAE		
	H_2_O	EtOH	EtOH:H_2_O (8:2)	H_2_O	EtOH	EtOH:H_2_O (8:2)	H_2_O	EtOH	EtOH:H_2_O (8:2)
Total colouring compound content (*C* betanin∙(mol·L^−1^))									
*Fruit*	0.008332^b^	0.01065^b^	0.01096^b^	0.009875^b^	0.01234^c^	0.01358^c^	0.009258^b^	0.01157^c^	0.01204^c^
Total colouring compound content (*C* chlorogenic acid ∙(mol·L^−1^))									
*Leaf*	0.001234^a^	0.002057^a^	0.002675^a^	0.008435^b^	0.01584^c^	0.02016^d^	0.00303^a^	0.005555^b^	0.006583^b^

Different superscripts letters indicate significant differences for parameters between the extraction methods at *p* < 0.05.

The best results in terms of TCC obtained for fruit and leaves, 0.01358 mol·L^−1^ and 0.02016 mol·L^−1^ respectively, both obtained for NE in E8, were intended for subsequent analysis and application.

### Compounds profile by LC–MS/MS analysis

Non-targeted LC–MS/MS analysis were performed only on the samples that resulted in higher yield in TCC and TPC values and detected 44 compounds, belonging to different chemical classes (Table 2). Polyphenolic compounds belonging to tannin, phenolic acid and anthocyanin families were identified.

**Table 2:** Metabolites identified in *R. coriaria* extracts by LC–MS/MS analysis.

ID	*R_t_ (min)	**R_t_ (min)	Compounds	*Fruit	**Leaf	[M + H]^+^	[M − H]^−^	Exact *m/z* Ratio	Accuracy (ppm)^a^	Main MS/MS fragments (m/z)	MSi status^b^
1	–	14.70	Trigalloyllevoglucosan		x	619.0903		619.0893	1.02	449; 279	2
2	–	19.88	di-O-galloyl-4,6-(S)-HHDP-scylloquercitol		x	771.1026		771.1022	0.44	431; 305	2
3	–	23.33	Trigalloyl-HHDP-scylloquercitol		x	923.1125		923.1116	0.88	771; 583; 305	2
4	–	25.91	Tetragalloyl-HHDP-D-scylloquercitol		x	1075.1249		1075.1248	0.15	923; 771	2
5	–	34.09	Pentagalloyl-HHDP-D-scylloquercitol		x	1227.1365		1227.1350	1.46	1175; 923	2
6	–	39.65	Hexagalloyl-HHDP-D-scylloquercitol		x	1379.1489		1379.1495	− 0.58	–	2
7	5.22	2.99	Galloylglucose	x	x		331.0671	331.0658	1.31	271; 169	2
8	22.02	11.34	Digalloylglucose	x	x		483.0761	483.0761	0.00	331; 271; 169	2
9	30.93	17.68	Trigalloylglucose	x	x		635.0884	635.0880	0.44	465; 483	2
10	37.91	20.95	Tetragalloylglucose	x	x		787.1001	787.1002	− 0.15	635; 617; 465	2
11	39.65	24.95	Pentagalloylglucose	x	x		939.1122	939.1113	0.88	769; 617; 169	2
12	43.29	27.64	Hexagalloylglucose	x	x		1091.1222	1091.1218	0.44	469	2
13	46.47	36.38	Heptagalloylglucose	x	x		1243.1331	1243.1319	1.17	545; 469	2
14	49.39	43.23	Octagalloylglucose	x	x		1395.1460	1395.1463	− 0.29	621; 545	2
15	34.11	20.41	Nonagalloylglucose	x	x		773.0740	773.0728	1.17	697; 621	2
16	38.19	21.84	Decagalloylglucose	x	x		849.0793	849.0794	− 0.15	773; 697; 621	2
17	11.82	–	Cyanidin-3-glucoside	x		449.1084		449.1077	0.29	287	2
18	10.09	–	Cyanidin-3-galattoside	x		449.1080		449.1090	− 0.58	287	2
19	12.59	–	7-methyl-cyanidin-3-galattoside	x		460.2486		460.2479	0.73	299; 298	2
20	27.68	16.79	Myricetin-rhamnoglucoside	x	x	627.1539		627.1519	2.04	481; 319	2
21	20.93	10.66	Myricetin-glucoside	x	x	481.0965		481.0947	1.75	319	2
22	23.28	12.06	Myricetin-glucuronide	x	x	495.0755		495.0759	− 0.44	319	2
23	13.87	6.02	Myricetin-ramnoside	x	x	465.1019		465.1012	0.73	319	2
24	17.75	6.83	Quercetin-glucoside	x	x	465.1019		465.1022	− 0.29	303	2
25	20.42	10.33	Quercetin-glucuronide	x	x	479.0810		479.0800	1.02	303	2
26	25.79	12.83	Rutin	x	x	611.1580		611.1598	− 1.75	465; 303	2
27	8.38	5.25	Kaempferol-glucoside	x	x	449.1069		449.1084	− 1.46	287	2
28	8.79	5.62	Quercetin-rhamnoside	x	x	449.1069		449.1069	0.00	303	2
29	7.00	3.89	Apigenin-glucoside	x	x	433.1122		433.1132	− 1.02	271	2
30	7.33	4.76	Kaempferol-rhamnoside	x	x	433.1122		433.1118	0.44	287	2
31	27.22	13.42	Myricetin-rhamnoside-gallate	x	x	617.1122		617.1123	− 0.15	471; 319	2
32	1.47	1.33	Quinic acid	x	x		191.0506	191.0509	− 0.29	173; 127; 85	2
33	22.99	11.56	Galloyl dihexoside	x	x		493.1197	493.1182	1.46	313; 271; 169	2
34	2.04	1.83	Ethyl-gallate	x	x	199.0601		199.0591	1.02	171; 153; 127	2
35	3.43	2.29	Digallic acid	x	x	323.0391		323.0392	− 0.15	153	2
36	19.07	8.45	Trigallic acid	x	x		473.0361	473.0364	− 0.29	321; 169	2
37	4.32	2.77	Galloylshikimic acid	x	x	327.0704		327.0704	0.00	153; 171	2
38	19.84	9.57	digalloilshikimic acid	x	x	479.0805		479.0812	− 0.73	305; 153	2
39	5.69	3.48	Ethyl-digallate	x	x	351.0705		351.0695	1.02	199; 153	2
40	24.14	12.55	Ethyl p-trigallate	x	x	503.0802/501.0688		503.0784/501.0670	1.75	457; 305; 153	2
41	32.57	19.16	Ethyl p-tetragallate	x	x		653.0796	653.0795	0.15	501; 349	2
42	2.80	2.07	Galloyl-glucoside	x	x	315.0705		315.0709	− 0.44	153	2
43	18.45	7.72	Galloylnorbergenin		x	467.0807		467.0806	0.15	297; 153	2
44	32.28	18.41	Digalloildiglucoside	x	x		645.1345	645.1360	− 1.46	493; 465; 313; 169	2

Average error (ppm) = 0.27.

Standard deviation (ppm) = 0.89.

^a^Error expressed in parts per million (ppm).

^b^MSI level of identification based on Sumner et al.^61^.

R_t_ = retention time; * = referred to fruit sample; ** = referred to leaf sample.

Of the 44 compounds identified, six were annotated as gallotannins: compound 1 was annotated as trigalloyllevoglucosan with a molecular ion (from now on m.i.) at m/z 619.0903 [M + H]^+^, while compounds 2 to 6 were identified as (poly)galloyl such as di-O-galloyl-4,6-(S)-HHDP-scylloquercitol (m.i. at m/z 771.1026 [M + H]^+^), trigalloyl-HHDP-scylloquercitol (m.i. at m/z 923.1125 [M + H]^+^), tetragalloyl-HHDP-D-scylloquercitol (m.i. at m/z 1075.1249 [M + H]^+^), pentagalloyl-HHDP-D-scylloquercitol (m.i. at m/z 1227.1365 [M + H]^+^) and hexagalloyl-HHDP-D-scylloquercitol (m.i. at m/z 1379.1489 [M + H]^+^), respectively. These compounds were exclusively found in the leaf extracts.

Likewise, ten glycosylated gallotannins were identified: compound 7 was annotated as galloylglucose with a m.i. at m/z 331.0671 [M − H]^−^ and fragment ions at 271 [M − H-60]^−^ and 169 [M − H-162]^−^ the latter corresponding to the loss of the glycosidic moiety, all other compounds, numbered 8 to 16 are (poly)galloyl glycosylated and identified as digalloylglucose with a m.i. at m/z 483. 0761 [M − H]^−^, trigalloylglucose with a m.i. at m/z 635.0884 [M − H]^−^, tetragalloylglucose with a m.i. at m/z 787.1001 [M − H]^−^, pentagalloylglucose with a m.i. at m/z 939.1122 [M − H]^−^, hexagalloylglucose with a m.i. at m/z 1091.1222 [M − H]^−^, heptagalloylglucose with a m.i. at m/z 1243.1331 [M − H]^−^, octagalloylglucose with a m.i. at m/z 1395.1460 [M − H]^−^, nonagalloylglucose with a m.i. at m/z 773.0740 [M − H]^−^ and decagalloylglucose with a m.i. at m/z 849.0793 [M − H]^−^, respectively. These compounds were found in both leaf and fruit extracts.

Fifteen anthocyanidins, flavonoids, and flavonols were identified, all glycosylated: compound 17 was annotated as cyanidin-3-glucoside with a m.i. m.i. at m/z 449.1084 [M + H]^+^ and fragment ion at 287 [M + H-162]^+^ corresponding to the loss of the glycosidic part; compounds 18 and 19 were annotated as cyanidin-3-galactoside, with a m.i. at m/z 449.1080, and as 7-methyl-cyanidin-3-galactoside, with a m.i. at m/z 460.2486, and both with fragment ion at 287 [M + H-162]^+^, the first, and 298 [M + H-162]^+^, the second, again corresponding to the loss of the glycosidic moiety. These compounds were exclusively found in the fruit extracts. While compounds 20 to 31 were identified as: myricetin-rhamnoglucoside with a m.i. at m/z 627,1539 [M + H]^+^ and fragment ions at 481 [M + H-146]^+^ and 298 [M + H-162]^+^ for the loss of the glycosidic moiety; myricetin-glucoside with a m.i. at m/z 481.0965 [M + H]^+^, myricetin-glucuronide with a m.i. at m/z 495.0755 [M + H]^+^ and myricetin-ramnoside with a m.i. at m/z 465.1019 [M + H]^+^, all with fragment ion at 319, [M + H-162]^+^, [M + H-176]^+^ and [M + H-146]^+^ respectively, all for loss of the glycosidic part; quercetin-glucoside with a m.i. at m/z 465.1019 [M + H]^+^ and quercetin-glucuronide with a m.i. at m/z 479.0810 [M + H]^+^, both with fragment ion at 303, [M + H-146]^+^ and [M + H-162]^+^ respectively; Rutin with a m.i. at m/z 611.1580 [M + H]^+^ and fragment ion at 465 [M + H-146]^+^ and 303 [M + H-162]^+^ for loss of the glycosidic part; kaempferol-glucoside with a m.i. at m/z 449.1069 [M + H]^+^ and fragment ion at 287 [M + H-162]^+^; quercetin-rhamnoside with a m.i. at m/z 449.1069 [M + H]^+^ and fragment ion at 303 [M + H-146]^+^; apigenin-glucoside with a m.i. at m/z 433.1122 [M + H]^+^ and fragment ion at 271 [M + H-162]^+^; kaempferol-rhamnoside with a m.i. at m/z 433.1122 [M + H]^+^ and fragment ion at 287 [M + H-146]^+^; myricetin-rhamnoside-gallate with a m.i. at m/z 617.1122 [M + H]^+^ and fragment ions at 471 [M + H-146]^+^ and 319 [M + H-162]^+^ due to loss of the glycosidic part. These compounds were exclusively found in the leaf extracts.

Ten compounds afferent to the organic acid family, such as gallic acid, comprising multiple units and esters were identified: compound 32 was annotated as quinic acid with a m.i. at m/z 191.0506 [M − H]^−^; compound 33 was annotated as galloyl dihexoside with a m.i. at m/z 493.1197 [M − H]^−^; compound 34 was annotated as ethyl gallate with a m.i. at m/z 199.0601 [M + H]^+^; compound 35 was annotated as digallic acid with a m.i. at m/z 323.0391 [M + H]^+^; compound 36 was annotated as trigallic acid with a m.i. at m/z 473.0361 [M − H]^−^; compound 37 was annotated as galloylshikimic acid with a m.i. at m/z 327.0704 [M + H]^+^; compound 38 was annotated as digalloylshikimic acid with a m.i. at m/z 479.0805 [M + H]^+^ compound 39 was annotated as ethyl-digallate with a m.i. at m/z 351.0705 [M + H]^+^; compound 40 was annotated as ethyl p-trigallate with a m.i. at m/z 503.0802/501.0688 [M + H]^+^; finally compound 41 was annotated as ethyl p-tetragallate with a m.i. at m/z 653.0796 [M − H]^−^. These compounds were exclusively found in the leaf extracts.

Finally, three gallotannins were identified: compound 42 was annotated as galloyl-glucoside with a m.i. at m/z 315.0705 [M − H]^−^ and fragment ion at 153 [M − H-162]^−^ due to the loss of the glycosidic moiety; compound 43 was annotated as galloylnorbergenin with a m.i. at m/z 467.0807 [M + H]^+^; compound 44 was annotated as digalloyldiglucoside with a m.i. at m/z 645.1345 [M − H]^−^ and fragment ions at 493 [M − H-152]^−^, at 465 [M − H-162-18]^−^ for loss of the glycosidic moiety and a water molecule at 313 [M − H-152-162-18]^−^ and at 169 [M − H-152-162]^−^. These compounds were exclusively found in the leaf extracts.

### Evaluation of staining values using the CIELAB method

Colouring measurements were made at random positions on three replicates of each sample type. The CIELAB colour coordinate values for the dyed fibres are given in Table 3 listed according to the fibres used in this study and according to the type of mordating employed.

**Table 3:** Partial colour differences (ΔL*, Δa* and Δb*), ‘chroma’ differences (ΔC*_ab_), ‘hue angle’ differences (Δh_ab_) and total colour difference (ΔE*_ab_) between the dyed fabrics.

Fruit	DWM(F)—U	DPL(F)—U	DPA(F)—U	DPL(F)—DWM(F)	DPA(F)—DWM(F)	DPL(F)—DPA(F)
Wool fibre						
ΔL*	31.38	25.00	27.00	6.38	4.38	2.00
Δa*	30.50	26.75	28.00	3.75	2.50	1.25
Δb*	21.38	18.88	20.00	2.50	1.38	1.13
ΔC*_ab_	36.19	31.71	33.38	4.49	2.82	1.67
Δh_ab_	35.00	34.09	34.19	0.91	0.82	0.10
ΔE*_ab_	48.70	41.19	43.74	7.81	5.22	2.62
Linen fibre						
ΔL*	17.14	17.61	13.62	8.87	3.52	3.99
Δa*	12.40	13.78	14.49	5.15	2.09	0.71
Δb*	15.08	18.59	15.14	2.51	0.06	3.44
ΔC*_ab_	17.97	21.72	19.02	3.76	2.70	2.71
Δh_ab_	7.43	6.78	11.20	0.65	4.41	4.41
ΔE*_ab_	25.98	29.08	24.99	10.56	4.09	5.32
Cotton fibre						
ΔL*	39.75	31.50	38.88	8.25	0.88	7.38
Δa*	41.63	35.75	41.00	5.88	0.63	5.25
Δb*	28.63	30.13	31.13	1.50	2.50	1.00
ΔC*_ab_	50.17	46.57	51.20	3.60	1.03	4.63
Δh_ab_	25.47	19.84	22.81	5.62	2.65	2.97
ΔE*_ab_	64.28	56.37	64.51	10.24	2.72	9.11

Leaf	DWM(L)—U	DPL(L)—U	DPA(L)—U	DPL(L)—DWM(L)	DPA(L)—DWM(L)	DPL(L)—DPA(L)
Wool fibre						
ΔL*	26.38	17.50	27.75	8.88	1.38	10.24
Δa*	1.38	2.63	5.38	1.25	4.00	2.75
Δb*	15.00	18.13	27.75	3.13	12.75	9.63
ΔC*_ab_	14.85	14.85	27.84	3.14	12.99	9.86
Δh_ab_	55.96	161.05	157.69	105.09	101.73	3.35
ΔE*_ab_	30.37	25.33	39.61	9.49	13.43	14.32
Linen fibre						
ΔL*	6.16	15.03	13.62	8.87	3.00	5.87
Δa*	11.52	16.67	14.49	5.15	6.18	1.03
Δb*	21.05	18.54	15.14	2.51	0.30	2.21
ΔC*_ab_	17.97	23.06	25.49	0.03	2.40	2.43
Δh_ab_	7.43	11.46	11.05	10.25	9.84	0.41
ΔE*_ab_	24.77	29.11	28.77	10.56	6.88	6.36
Cotton fibre						
ΔL*	33.50	18.50	31.25	15.00	2.25	12.75
Δa*	0.38	2.88	5.38	2.88	5.00	2.50
Δb*	22.38	22.50	36.75	0.13	14.38	14.24
ΔC*_ab_	22.03	22.03	36.61	0.17	14.58	14.41
Δh_ab_	25.47	146.83	146.62	172.29	171.08	1.21
ΔE*_ab_	40.29	29.27	48.54	15.27	15.39	19.28

F = fruit. L = leaf. U = Undyed. DWM = stained with extract dye without the use of mordants. DPL = pre-treatment with lemon juice. DPA = pre-treatment with aluminum potassium sulphate.

**Figure 2:**
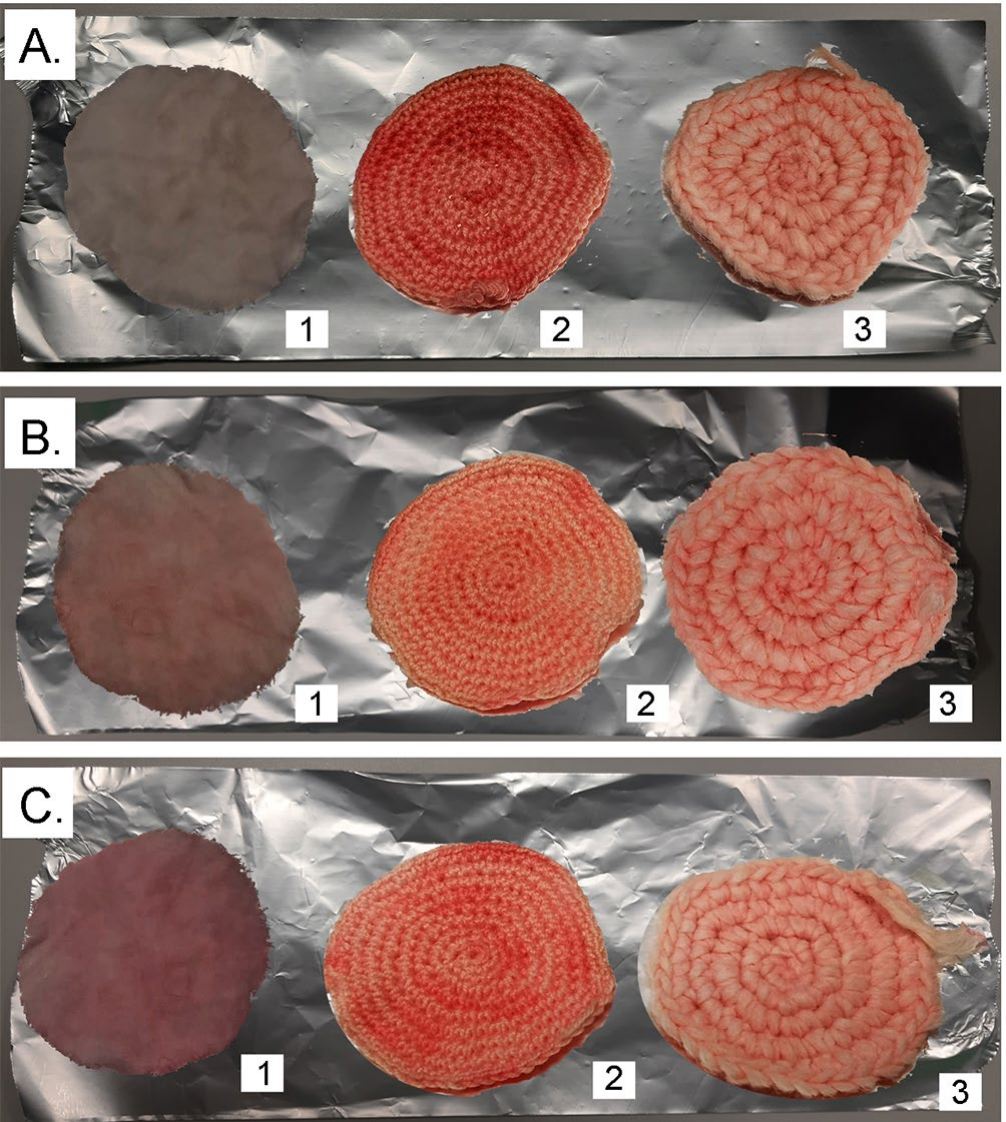
Fibre discs of (1) linen, (2) cotton and (3) wool after treatment with dye solutions of *R. coriaria* fruits: (**A**) DWM; (**B**) DPL; (**C**) DPA.

From an initial analysis of the data regarding the colouring performed with the dye obtained from the fruits of *R. coriaria* (Supplementary Table 3 and Fig. 2), it is possible to examine what the various parameters express with respect to the CIELAB coordinates recorded. The parameter L* (expressing colour brightness) has a value ranging between 53.50 and 59.90 CIELAB units for samples obtained from the dyeing of wool fibres; for samples of dyed linen fibres L* varies between 73.90 and 77.90 CIELAB units; while for samples of dyed cotton fibres L* varies between 48.00 and 56.25 (all samples, identified as undyed fabric U, had 84.88, 91.50 and 87.75 CIELAB units as the L* control value for wool, linen and cotton fibres, respectively).

As for the value of a*, which is associated with the transition from green ( −) to red ( +), it varied in a data range from 1.25 (U) to 31.75 (DWM), 28.00 (DPL), 29.25 (DPA), for wool fibre, in a data range from − 0.25 (U) to 12.15 (DWM), 13.53 (DPL), 14.24 (DPA), for flax fibre and from 1.38 (U) to 43.00 (DWM), 37.12 (DPL), 42.37 (DPA), for cotton fibre. The quantification of these data verifies the coloration taken on by the fibres, which took on a pinkish colour.

The b* value, which is closely associated with the transition from blue ( −) to yellow ( +), is in a data range from 4.87 (U) to 26.25 (DWM), 23.75 (DPL), 24.88 (DPA), for wool, in a data range of 9.00 (U) to 24.08 (DWM), 27.58 (DPL), 24.14 (DPA), for flax fibre and 2.62 (U) to 31.25 (DWM), 32.75 (DPL), 33.75 (DPA), for cotton fibre. In this case, the positive parameter presents in the acquired data stands for the shift toward a more purplish colour.

For the parameter C*_ab_, corresponding to colour saturation, variation is observed in a data range of 5.07 (U) to 41.26 (DWM), 36.77 (DPL), 38.44 (DPA), for wool fibre, in a data range of 9.03 (U) to 26.97 (DWM), 30.73 (DPL), 28.02 (DPA), for linen fibre and for cotton fibre in a data range that is between 3.00 (U) and 53.17 (DWM), 49.57 (DPL), 54.20 (DPA). Looking at the variation from the sample fibres, in all three cases, there was an increase in the value: for wool above 30–35 CIELAB units, for linen above 17–21 CIELAB units, and for cotton above 46–51 CIELAB units: this means that the colour saturation is quite pronounced and this denotes effective dyeing.

About the dyeing angle, with h_ab_ parameter, for all dyed fibres, with and without etchant, an angle between 39.93° and 40.84° (DWM), between 59.46° and 63.87° (DPL) and between 36.07° and 41.69° (DPA) was obtained, i.e. one was in the pink-purple dyeing zone.

**Figure 3:**
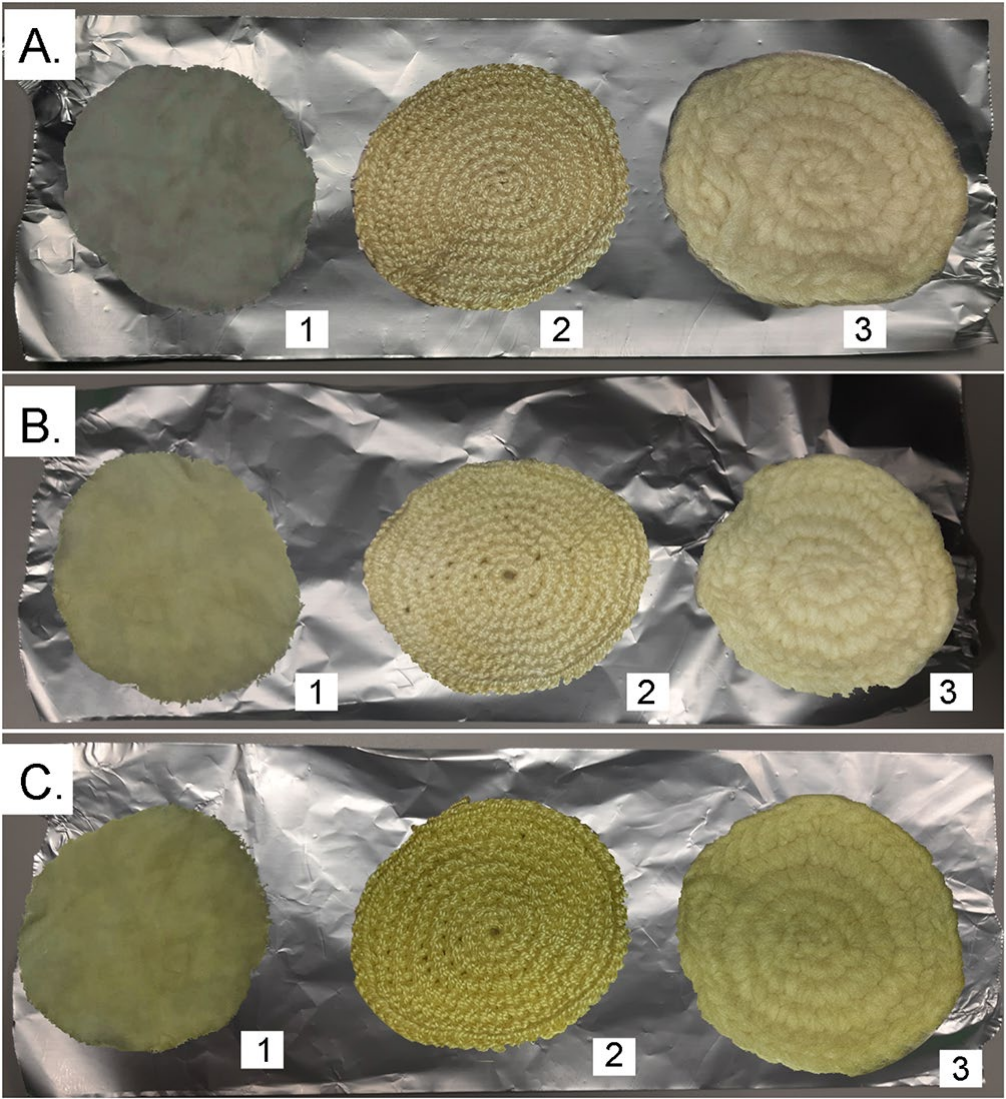
Fibre discs of (1) linen, (2) cotton and (3) wool after treatment with dye solutions of *R. coriaria* leaves: (**A**) DWM; (**B**) DPL; (**C**) DPA.

Concerning data on colouring with the dye obtained from *R. coriaria* leaves (Supplementary Table 3 and Fig. 3), it is possible to make different considerations with respect to the recorded CIELAB coordinates. The L* parameter has a variation between 84.88 (U) to 58.20 (DWM), 67.37 (DPL), 57.13 (DPA), for wool fibre, in a data range from 91.50 (U) to 85.34 (DWM), 76.47 (DPL), 82.34 (DPA), for flax fibre and from 87.75 (U) to 54.25 (DWM), 69.25 (DPL), 56.50 (DPA), for cotton fibre.

As regards the value of a* it varies in a data range from 1.25 (U) to − 0.13 (DWM), − 1.38 (DPL), − 4.13 (DPA), for wool fibre, in a range of data from − 0.25 (U) to 11.27 (DWM), 16.42 (DPL), 17.45 (DPA), for flax fibre and from 1.38 (U) to 1.00 (DWM), − 1.50 (DPL), − 4.00 (DPA), for cotton fibre. The quantification of this data verifies the colouring taken on by the fibres, which have taken on a yellowish colour.

The b* value is included in a data range between 4.87 (U) and 19.88 (DWM), 23.00 (DPL), 32.63 (DPA), for wool, in a data range between 9.00 (U) and 30.05 (DWM), 27.54 (DPL), 29.75 (DPA), for flax fibre and between 2.62 (U) and 25.00 (DWM), 25.13 (DPL), 39.37 (DPA), for cotton fibre. Also in this case, the positive parameter presented in the acquired data indicates the shift towards a darker purple colour.

For the C*_ab_ parameter, a variation is observed in a data range between 5.07 (U) and 19.91 (DWM), 23.05 (DPL), 32.91 (DPA), for wool fibre, in a data range of 9.03 (U) to 32.09 (DWM), 32.06 (DPL), 34.49 (DPA), for flax fibre and for cotton fibre in a data range between 3.00 (U) and 25.03 (DWM), 25.20 (DPL), 39.61 (DPA). The variation compared to the sample fibres, in all three cases, is positive: for wool above 15–28 CIELAB units, for linen above 23–25 CIELAB units and for cotton above 22–36 CIELAB units: therefore, the saturation of colour is important and corresponding to an effective dye.

As regards the h_ab_ angle, it is observed that for all dyed fibres, with and without mordant, an angle between − 86.76° and 18.97° (DWM), between 59.20° and 69.44° (DPL) and between − 87.00° and − 84.08° (DPA), i.e. one was in the yellow-beige dye zone.

Statistical analysis performed on the colour parameters recorded, revealed important differences between the treatments (Supplementary Tables 3–4). The uncoloured (U) fibre samples have different parameters from the coloured samples, this indicates that the determination of colorimetric parameters did not result in bias. In addition, the parameters analysed for samples coloured with extract without the use of mordant (DWM) were found to be different from those where the mordant was used, both in the pretreatment with lemon juice (DPL) and in the pretreatment with aluminium potassium sulphate (DPA). Differences are reported in Supplementary Tables 7–8.

A final assessment was made for dye's resistance to washing practices and light exposure^34–36^. (Supplementary Tables 5–6). All dye results can be seen in Figs. 2, 3.

## Discussion

The opportunity to replace synthetic dyes (known to be a major source of environmental pollution) with natural ones^37^ is very interesting and feasible. Using traditional plants, but also SRMs, is nowadays of primary interest to meet the goals of Agenda 2030. Their use would benefit both from the perspective of circular economy and sustainability to reduce environmental impacts. This study showed that *R. coriaria* embraces sustainability in textile processes, representing a viable and environmentally friendly alternative to traditional dyes.

Looking at the values obtained, we can assess that the soluble solid content (expressed in degrees Brix, °Bx), is low compared with the specific gravity of each extract (the value of extract weight expressed in grams of extract per grams of dry matrix weight), as already verified in other works^38–41^. This result, according to total colorant content analysis (TCC) and total phenolic compound analysis (TPC), would suggest that the largest percentage of extract weight consisted of colorant compounds and phenolic compounds^42,43^.

The use of different techniques and, of NE, showed the opportunity for scalability of the process. This application would allow various companies operating in the textile sector to obtain molecules from plants, to be used as natural dyes. The results outlined that the optimal extraction conditions with NE appear to be great, with better times and performance, compared with ME and UAE. In fact, NE turns out to be the technique that is most successful in obtaining TPC in amounts almost twice as high as ME, both in fruit and leaf extracts; while in the comparisons of UAE, first considering fruits, the extracts are comparable, except in the case of the E8, where the extract with NE turns out to be 25% higher, considering instead leaves, in all cases the extracts with NE are 25–35% higher. In addition, considering equal conditions for solvent, extraction time and ratio between quantity of solid and solvent in the extraction phase, we observed a significant increase in terms of TPC and TCC compared to the dry residue. NE allowed us to obtain more performing extracts compared to the other techniques considered, because it carries out an active and not passive extraction (as in the case of ME): with the same solvent, both for the H1 and E8, the NE is better than the ME and UAE. Regarding the absorption spectra (Supplementary Figs. 1–2), elaborated from the characterization of the TCCs obtained from both fruit and leaves, it is seen that also in this case E8 is the best in the extraction of colouring compounds. The NE technique also in this case appears to be the most appropriate in obtaining TCCs. Overall, the results obtained allowed us to state that NE technique, with a mixture of eco-compatible solvents, is a valid application to obtain natural colouring solutions.

TCC and TPC results were evaluated to identify the extract with the higher colouring potential (maximum TCC). This was used for subsequent fibre colouring. These best extracts (one for leaves and one for fruits) were subjected to LC–MS/MS analysis. The compounds identified in sumac extracts submitted for analysis, based on accurate mass determination of deprotonated/protonated ions obtained from MS data and MS/MS fragmentation pattern, as well as other relevant literature information yielded a result (Table 2) among colouring phenolic components from various families.

LC–MS/MS analysis were performed in positive or negative mode as needed to obtain better visualization and/or fragmentation of individual analytes as needed. In the literature there are several references to the use of analyses performed in positive or negative mode, even on different matrices, to obtain qualitative-quantitative data on some compounds of interest such as organic acids or glycosidic flavonoids. In the example for compounds **1** (see ID in Table 2) a positive mode analysis was performed as reported in El-Sayed et al.^44^. While in Carini et al.^45^, analytes **27** and **29** were identified with positive mode analyses. Also, for analytes **21**, **23**, **28** and **30**, in the work of Kiehne et al.^46^, the analysis was performed and obtained in positive mode. The compound **17** and **18** were identified with positive mode analyses as reported in Määttä al.^47^.

In this context, the data obtained qualitatively indicate that sumac is an abundant source of colouring compounds (tannins and anthocyanins) that can be employed as an alternative to synthetic dyes if supported by an established technological application.

Depending on the staining technique, we obtained different results and compared the different staining levels obtained on the different fibre discs. The colouring in some cases was very effective, for example in the case of the colouring obtained with the leaf extract for DPA (L) and in the case of the colouring obtained with the fruit extract for both DWM (F) and DPA (F).

From all the measurements carried out on the fibres coloured with both fruit and leaf extracts of *R. coriaria*, it can be observed that the colouring extracts lead to a specific colour (yellow and red) based on properties of the components present, and it is also influenced by the mordants and the fibre used, with respect to the depth and tones of the colour, as reported in the literature^48–50^: the molecular interactions between mordants, colouring compounds and fibres are based on the chemical bonds established^51^.

In particular, the following observations can be made regarding the two colouring extracts applied and the mordants used. From Table 3 it is possible to see the differences that exist between the samples obtained: precisely the absorption of the dye is compared, considering the type of dyeing done (with or without the use of mordant). Considering these parameters, between wool, linen and cotton, this absorption is greater for cotton, which seems to be a good support textile for dyes obtained from *R. coriaria* fruit and leaf. As regards the evaluation of the use of mordant or its non-use, DPL on linen and cotton is more important for fruit dye compared to DWM and DPA; compared to the leaf dye, all applications with mordant, DPL and DPA, were found to be valid, while between DPL and DPA, the second had a slightly better application.

Comparing with the literature, considering the work done by Mikropoulou E. et al.^52^, different considerations can be taken on cotton and wool in DWM and DPA cases, including in the evaluation of ΔL*, ΔC*_ab_, Δh_ab_ and ΔE*_ab_ data. Regarding the stained samples without etchant (DWM): leaf extracts of *R. coriaria*, in the evaluation of the parameters L*, C*_ab_, h_ab_, compared with plants such as *Crocus sativus* L., *Curcuma domesica* Valet., *Reseda luteola* L., *Rhamnus saxatilis* Jacq. subsp. *tinctoria* and *Rhamnus lycioides* subsp. *oleoides* L., it can be seen that for cotton, a result (54.25, 25.03 and 87.00) was obtained very close to the average of the values (79.99, 26.90 and 83.75) obtained in the work counting the different species; for wool, however, a result (58.50, 19.91 and 18.97) was obtained below the average of the values (63.80, 43.95 and 77.29) obtained in the work. This assessment can be effective by evaluating the yellow colouring of the fibres.

Regarding the extracts of *R. coriaria* fruits, in the evaluation of the parameters L*, C*_ab_, h_ab_, compared with plants such as *Carthamus tinctorius* L., *Alkanna tinctoria* Tausch., *Rubia tinctorum* L., *Kermes vermilio* Planchon, *Dactylopius coccus* O. Costa, *Caesalpinia sappan* L. and *Caesalpina echinata* Lam., again in the work done by Mikropoulou E. et al.^52^, it can be observed that for the stained samples without etchant (DWM): for cotton a result (48.00, 53.17 and 36.07) was obtained that was significantly different from the average of the values (67.16, 16.30 and 131.98) obtained in the work counting the different species; on the other hand, for wool a result (53.50, 41.26 and 39.93) was obtained that was discreetly better than the average of the values (41.05, 31.47 and 41.04) obtained in the work. This assessment can be effective by evaluating the red colouring of the fibres.

Regarding linen and wool, the work of Toussirot M. et al.^53^, where the extraction method and the dye application technique to the fibres are very similar, using *Hubera nitidissima* (Dunal) Chaowasku as matrix, it is reported that for L*, C*_ab_, h_ab_ (obtained from a* and b* values) values of 58.98, 43.47 and − 87.91, the yellow colour obtained from *R. coriaria* leaves and applied to wool has close values for L* (58.50) while for C*_ab_ and h_ab_ the values are very divergent (19.91 and 18.97) with significant improvement of the same; on the other hand, for linen, considering the same values (L*, C*_ab_, h_ab_) for *R. coriaria* values of 91.50, 9.06 and − 20.66 were obtained, which when compared with 88.76, 20.36 and − 82.32, have a similar and therefore comparable trend.

Looking at the colour enhancing parameters, as regards the colours carried out with fruit extracts, the most interesting variation of the ΔL* parameter (Table 3) has a value of 39.75 CIELAB units for DWM on cotton, which indicates an important and perceptible colour variation in the brightness of the fibre due to the action of the dye. The h_ab_ value represents the main attribute of colour perception, therefore the results obtained show us that the use of the mordant, whether DPL or DPA, had no significant altering effects on the chromatic values.

As previously, in the evaluation of the parameters ΔL*, ΔC*_ab_, Δh_ab_ and ΔE*_ab_, compared with the work of Mikropoulou E. et al.^52^, the following considerations can be made: for cotton in the use of *R. coriaria* leaves, the values of Δ (2.25, 14.58, − 171.08 and 15.39) are very different to the average values measured for the other species (− 2.46, 7.93, 1.71 and 9.04) that colour yellow; in the use of *R. coriaria* fruits, again for cotton the values of Δ (0.88, 1.03, 2.65 and 2.72) are below the average of the values measured for the other species (2.77, 6.85, 6.33 and 22.23) that colour red. Regarding wool in the use of *R. coriaria* leaves, as in the previous case, the values of Δ (− 1.38, 13.00, − 101.73 and 13.43) are very close to the average values measured for the other species (0.61, 9.70, 1.74 and 7.95) that colour yellow; in the use of *R. coriaria* fruits, again for cotton the values of Δ (4.38, − 2.82, 0.82 and 5.22) are very close the average of the values measured for the other species (5.61, 3.37, 3.78 and 7.88) that colour red.

In conclusion, the possibility to obtain colouring compounds from *R. coriaria* by using alternative techniques and methodologies like NE, could be advantageous for both small and medium-sized enterprises that want to apply innovation technology to consolidated but not cutting-edge processes, also obtaining advantages from sustainability point of view.

## Materials and methods

### Chemicals and reagents

All solvents and reagents were of high quality with a high grade of purity. Bidistilled water was used for the preparation of the solutions and extraction of matrices. All extracts were used without any purification and all the solutions analysed, where necessary, were prepared by diluting the stock solutions of the extracts in water or in a hydroalcoholic solution. Folin-Ciocȃlteu reagent, Ethanol ≥ 99.9% ACS for analysis, acetonitrile hypergrade for LC–MS, hydrochloric acid 37% RPE for analysis, potassium bicarbonate, anhydrous sodium carbonate for analysis, gallic acid ACS for analysis, betanin and chlorogenic acid were acquired from Sigma-Aldrich Chemical Company (Milano, Italy) and from Merck (Milano, Italy).

### Plant materials: recovery and storage

Fruits and leaves were sampled from plants placed in different sites in the province of Palermo, (37°56′ 22" N 13°59′ 01" E) within the municipalities of Isnello and Collesano. The soils on which the plants grew were acidic and basic in nature.

Conservation status of *Rhus coriaria* L. according to the IUCN is Least Concern (LC)^54^, so it is not subject to protection or legislative constraints regulating its removal from the wild. The samples were collected without breaking any laws and in accordance with the guidelines of the IUCN Policy Statement on Research Involving Species at Risk of Extinction^55^.

The voucher specimens (PAL-73607) were deposited at the Herbarium Mediterraneum Panormitanum of the botanical garden of the University of Palermo. Rosario Schicchi e Anna Geraci identified all samples.

The collected samples were immediately vacuum sealed in clean paper bags and stored in refrigerated boxes at 4 °C; the samples were transported to the laboratory and subsequently, they were carefully washed with distilled water to remove impurities. Before proceeding with the extractions, the samples were dried and subsequently chopped manually to a previously chosen size.

### Preparation of *R. coriaria* extracts

The extractions were performed in triplicate for both matrices: three different solid–liquid extraction techniques, maceration (ME), Extractor Naviglio® (NE)^32^ and ultrasound-assisted (UAE) were used. The solvents used for the extractions were two, ethanol (EtOH or E1) and double-distilled water (H_2_O or H1), plus a mixture of them, a solution of EtOH and H_2_O in a ratio of 8:2 (v/v) (E8). All extractions were made at a room temperature of 25.00 ± 1.00 °C, for a time equal to 1 h. In general, to minimize the possible degradation of unstabilized dye molecules, dark containers were used, including for storage (taken place in refrigerators at − 20 °C for a few days before submitting the samples for analysis). Samples obtained after extraction were filtered to make them clear (Supplementary Fig. 3). All instruments, instrument settings and method explication are reported in Supplementary Table 7.

All the extracts were subjected to removal by evaporation of the solvent (through a HEIDOLPH Heizbad Hei-Vap rotary evaporator by Schwabach from Germany) to be able to analyse them and subsequently use them as dyes.

### Determination of analytical parameters

For all the extracts, various analytical parameters were quantified: the pH, the soluble solid residue, the dry residue. Total phenolic content was calculated using the Folin-Ciocȃlteu reagent and the total colouring content was calculated. All samples subjected to analysis were resolubilized in a standard solution of H_2_O and EtOH with a 95:5 (v/v) ratio.

With reference to parameters such as dry residue and total extraction yield (Residue and Extract, respectively, in Supplementary Table 1), they have been reported for each extract obtained with the various techniques and solvents; the dry residue allows extrapolation of the amount of sample extracted from the matrix per amount of solvent used, then also calculates the extractive power for the type of solvent used, depending on the technique; total extraction yield calculates the maximum amount of the extract obtained per amount of matrix used revealing the maximum amount of substance that can be extracted with that specific method under the conditions listed.

#### Soluble solid contents (°Bx) and pH

°Bx, pH and temperature were measured for all extracts through a Brix and Gravity Refractometer and a CRISON GLP21 pH meter (L'Hospitalet de Llobregat, Barcelona, Spain). Considering Supplementary Table 1, parameters referring to pH to evaluate the acidity-basicity of the samples and to °Bx to evaluate the soluble solids content by measuring the content of dissolved carbohydrates in solution relating in this case precisely to the dissolved solids content are made explicit.

#### Total phenolic compound content (TPC)

A Spectroquant® Pharo 300 UV/Vis spectrophotometer of MERCK (Milano, Italy) equipped with a 1.0 cm long optical path glass cell was used for all absorbance measurements.

TPC was measured according to the Folin-Ciocȃlteu reagent method^56^, applied with some modifications as in other works^33^: 5 μL of sample extract, 125 μL H_2_O and EtOH solution in ratio 95:5 (v/v), 120 μL of Folin-Ciocȃlteu reagent and 1.25 mL of Na_2_CO_3_ (7.5% w/v); the absorbance at 760 nm was measured and TPC was expressed as the concentration of gallic acid equivalents (GAE) in mol GAE∙L^−1^.

#### Total colouring compound content (TCC)

Extracts of *R. coriaria* were characterized colorimetrically by UV–Vis spectrophotometry after appropriately dilution^57^. UV–Vis spectra were acquired in the visible region of 300–800 nm. The following were used as standards for the determination of the colouring compounds: a betanin standard extracted from red beet diluted with dextrin (for fruit extract); a chlorogenic acid standard (for leaf extract). Calibration curves were prepared for the standards using final concentrations of 0.015, 0.032, 0.065, 0.130, 0.25, 0.5 and 1.0 mg∙mL^−1^ in triplicate.

### LC–MS/MS qualitative analysis

*R. coriaria* fruit and leaf extracts selected (based on the best values of TCC and TPC obtained) for fibre staining were subjected to LC–MS/MS analysis through a Thermo Scientific UltiMate 3000 UHPLC and with a Q Exactive™ Hybrid Quadrupole-Orbitrap™ High Resolution Mass Spectrometry (Thermo Fischer Scientific Inc., Darmstadt, Germany) equipped with an electrospray ionization source (ESI) operating by switching between the positive and negative ionization modes. The chromatographic system uses a Luna® C18 150 × 2 mm, 3 µm (100 Å) column (Phenomenex®, Castel Maggiore, Bologna, Italy); the mobile phase used consisted of: 0.1% (v/v) formic acid in water (eluent A) and acetonitrile (eluent B); the gradient, linear type, was from 5 to 50% of eluent B in 30 min with a flow rate of 0.2 mL∙min^−1^. The mass spectrometer scan was performed in both positive and negative mode identifying the metabolites in (I) based on the measurement of their accurate mass, in (II) based on the acquired MS/MS spectra, in (III) based on the comparison with literature data and, if available, in (IV) based on comparison with pure standards. Other following instrumental parameters were used: capillary temperature sets at 320 °C, source temperature sets at 380 °C, flow rate of sheath gas and auxiliary gas (nitrogen gas N_2_) set at 9 L∙min^−1^; capillary voltage was ± 4 kV, while the fragmentation voltage varied from10 to 20 eV to fragment [M + H]^+^ ions, and from 80 to 120 eV in the case of [M + Na]^+^ions.

To obtain qualitative information on colouring components extracted from fruits and leaves of *R. coriaria*, a non-targeted LC–MS/MS analysis was performed in both positive and negative modes. Since no commercial standards were available for all sumac phenols and phytochemical compounds detected in this work, they were characterized through the MS data, along with the interpretation of the obtained MS/MS spectra against those found in literature. Identification was also conducted through phytochemicals previously identified, belonging to the same botanical family or species, or also consulting the public databases SciFinder Scholar (https://scifinder.cas.org), Kegg Ligand Database (http://www.genome.jp/kegg/ligand.html), ChemSpider (http://www.chemspider.com), NIST Chemistry WebBook (https://webbook.nist.gov/chemistry/) and Phenol-Explorer (www.phenol-explorer.eu).

### Dyeing method

Fibres such as cotton, wool and linen were selected for the study. Fabric discs (Supplementary Fig. 4) with a diameter of 6.5 cm were prepared from available fibres to have complementary area units, and their weight parameters were recorded. The applied methodology followed the work adopted by Scarano et al.^33^ (Supplementary Figs. 5–6). Following the reported method, the fabrics were drained and dried and subsequently washed twice (for approximately 2 min) at 40 °C with distilled water with a neutral detergent, repeating the colouring and washing process 3 times and performing the experiments in triplicate. Unstained fibre samples were used as controls (Supplementary Fig. 4). The extract used for colouring had a concentration of colouring molecules equal to 0.5 g∙mL^−1^.

### Calculation of colorimetric and fastness properties

The dyed fabric samples were analysed with a NR100 colorimeter from 3NH Technology Co., LTD, consisting of a flat viewing aperture of 8 mm in diameter and an angular viewing aperture of 4 mm in diameter. All measurements concerned the samples subjected to staining, using in part the method applied by Chan-Bacab et al.^58^, and Alessi et al.^59^, where CIELAB fibre and data analysis standards are used.

Colour measurement parameters were recorded according to CIELAB coordinates, L*, a*, b*, C*_ab_ and h_ab_. Each total measurement was carried out on an average of eight coloration samplings: minimum reading parameter defined by the fixed section of the inverted exponential decay graph^60^.

### Evaluation method for staining values

Colour fastness, which characterizes the resistance of a material's colour to fading or running, is directly proportional to the bonding strength between the dye and the fibre. Testing the colour fastness of textiles is prioritized over other tests using harsh chemicals (such as bleach) or other more specific cleaning products. Both lightfastness and fastness to washing were evaluated and both on a scale of 1 to 8 (a higher number indicates better fastness). The ratings shown in Supplementary Tables 5–6 translate the colour change measured through the standard test for the colour fastness to artificial light (xenon arc lamp)^36^ of the dyed material: this parameter is evaluated by comparing the colour deterioration of the sample with that dyed and not subjected to deterioration by calculating the ΔL*, Δa*, Δb*, ΔC*_ab_, Δh_ab_ and ΔE*_ab_. These Δ are then translated to the scale represented in Supplementary Tables 5–6.

### Data analyses

Data were shown as mean and standard deviation, SD (triplicate measures). For the statistical analysis of the data, first the normal distribution of data was checked by the Shapiro–Wilk test. Some variables of CIELAB colour coordinates (L*, a*, b*, C*_ab_ and h_ab_) violate normality, so a Kruskal–Wallis non-parametric test was used to verify if there were differences between the different fibre types. In order to determine which groups were different from others, post-hoc Dunn (*p* < 0.05) test was conducted. For normal distributed data, differences of parameters (pH, Brix, Residue, Extract) for each solvent (H_2_O, EtOH, EtOH:H_2_O (8:2)) between the four extraction methods (ME, NE and UAE) were detected using one-way ANOVA with Tukey's post-hoc test (*p* < 0.05).

### Ethical approval and consent to the publication

The authors state that this research does not require ethical consent. The authors declare their consent to participate. The authors declare their consent to the publication.

### Supplementary Information

Supplementary Information.

## References

[1] Verma, M. & Stone, J. The Value of Air Monitoring. 1–3

[2] Angelini LG, Pistelli L, Belloni P, Bertoli A, Panconesi S (1997). *Rubia tinctorum* a source of natural dyes: Agronomic evaluation, quantitative analysis of alizarin and industrial assays. Ind. Crops Prod..

[3] Prigioniero A (2020). Ethnobotany of dye plants in Southern Italy, Mediterranean Basin: Floristic catalog and two centuries of analysis of traditional botanical knowledge heritage. J. Ethnobiol. Ethnomed..

[4] Patra A, Abdullah S, Pradhan RC (2022). Review on the extraction of bioactive compounds and characterization of fruit industry by-products. Bioresour. Bioprocess.

[5] Reguengo LM, Salgaço MK, Sivieri K, Maróstica Júnior MR (2022). Agro-industrial by-products: Valuable sources of bioactive compounds. Food Res. Int..

[6] United Nation. Transforming our World: The 2030 Agenda for Sustainable Development. in vol. A/RES/70/1 (2015).

[7] Scarano P, Sciarrillo R, Tartaglia M, Zuzolo D, Guarino C (2022). Circular economy and secondary raw materials from fruits as sustainable source for recovery and reuse. A review. Trends Food Sci. Technol..

[8] European Commission. Closing the Loop - An EU action plan for the Circular Economy. in *Communication from the Commission to the European Parliament, the Council, the Europena Economic and Social Committee and the Committee of the Regions* 1–21 (2015).

[9] European Commission. New Circular Economy Strategy - Environment - European Commission. https://ec.europa.eu/environment/circular-economy/index_en.htm (2020).

[10] Wu T (2013). Evaluation of antioxidant activities and chemical characterisation of staghorn sumac fruit (*Rhus hirta* L.). Food Chem..

[11] Gulmez M, Oral N, Vatansever L (2006). The effect of water extract of sumac (*Rhus coriaria* L.) and lactic acid on decontamination and shelf life of raw broiler wings. Poult. Sci..

[12] Abdallah S, Abu-Reidah I, Mousa A, Abdel-Latif T (2019). *Rhus coriaria* (sumac) extract reduces migration capacity of uterus cervix cancer cells. Revista Brasileira de Farmacognosia.

[13] Nasar-Abbas SM, Halkman AK (2004). Antimicrobial effect of water extract of sumac (*Rhus coriaria* L.) on the growth of some food borne bacteria including pathogens. Int. J. Food Microbiol..

[14] Candan F (2008). Effect of *Rhus coriaria* L. (Anacardiaceae) on superoxide radical scavenging and xanthine oxidase activity. J. Enzyme Inhib. Med. Chem..

[15] Mirian M, Behrooeian M, Ghanadian M, Dana N, Sadeghi-Aliabadi H (2015). Cytotoxicity and antiangiogenic effects of *Rhus coriaria*, *Pistacia vera* and *Pistacia khinjuk* oleoresin methanol extracts. Res. Pharm. Sci..

[16] Abu-Reidah IM, Ali-Shtayeh MS, Jamous RM, Arráez-Román D, Segura-Carretero A (2015). HPLC–DAD–ESI-MS/MS screening of bioactive components from *Rhus coriaria* L. (Sumac) fruits. Food Chem..

[17] Nozza E (2020). *Rhus coriaria* L. fruit extract prevents UV-A-induced genotoxicity and oxidative injury in human microvascular endothelial cells. Antioxidants.

[18] Romeo FV (2015). Chemical characterization of different sumac and pomegranate extracts effective against botrytis cinerea rots. Molecules.

[19] Bursal E, Köksal E (2011). Evaluation of reducing power and radical scavenging activities of water and ethanol extracts from sumac (*Rhus coriaria* L.). Food Res. Int..

[20] Chakraborty A (2009). DNA-protective effects of sumach (*Rhus coriaria* L.), a common spice: Results of human and animal studies. Mutation Res./Fundamental Mol. Mech. Mutagenesis.

[21] El Hasasna H (2015). *Rhus coriaria* induces senescence and autophagic cell death in breast cancer cells through a mechanism involving p38 and ERK1/2 activation. Sci. Rep..

[22] El Hasasna H (2016). *Rhus coriaria* suppresses angiogenesis, metastasis and tumor growth of breast cancer through inhibition of STAT3, NFκB and nitric oxide pathways. Sci. Rep..

[23] Falcão L, Araújo MEM (2013). Tannins characterization in historic leathers by complementary analytical techniques ATR-FTIR, UV-Vis and chemical tests. J. Cult. Herit..

[24] Kurucu S, Koyuncu M, Güvenç A, Baser KHC, Özek T (1993). The essential oils of *Rhus coriaria* L. (Sumac). J. Essential Oil Res..

[25] Hassanp S (2011). Plants and secondary metabolites (Tannins): A review. Int. J. Forest Soil Erosion.

[26] Sakhr K, El Khatib S (2020). Physiochemical properties and medicinal, nutritional and industrial applications of Lebanese Sumac (Syrian Sumac - *Rhus coriaria*): A review. Heliyon.

[27] Hamdy DM, Hassabo AG (2021). Various natural dyes from different sources. J. Textiles, Coloration Polym. Sci..

[28] Baaka N (2022). Sumac (*Rhus Tripartita*): A natural dye used for simultaneous coloration and functional finishing on textiles. J. Nat. Fibers.

[29] Ben Mahmoud S (2015). Characterization of sumac (*Rhus tripartitum*) root barks tannin for a potential use in wood adhesives formulation. Wood Sci. Technol..

[30] Ayanoglu F, Caliskan O, Bayazit S, Kocer O (2023). Traditional medicinal and aromatic trees in Türkiye: Laurel (*Laurus nobilis* L.), Sumac (*Rhus coriaria* L.), Hawthorn (*Crataegus* spp.) and Carob (*Ceratonia siliqua* L.). Med. Aromatic Plants Turkey.

[31] Indi, Y. M., Patil, P. D. & Jujare, T. D. Studies in Natural Dyeing (Part 2). *DYE CHEM PHARMA BUSINESS NEWS* 42–44 (2016).

[32] Naviglio D (2003). Naviglio’s principle and presentation of an innovative solid-liquid extraction technology: Extractor Naviglio®. Anal. Lett..

[33] Scarano P (2022). Recovery and valorization of bioactive and functional compounds from the discarded of *Opuntia ficus-indica* (L.) Mill. Fruit Peel. Agron..

[34] Janani L, Winifred D (2013). Suitability of dyes from mulberry and coffee leaves on silk fabrics using eco-friendly mordants. Int. J. Sci. Res. Public..

[35] Singh SV, Purohit MC (2014). Evaluation of colour fastness properties of natural dye extracted from *Symplocos racemosa* (Lodh) on wool fibres using combination of natural and synthetic mordants. Indian J. Fibre Text Res..

[36] Oger B (1996). Fastness to light and washing of direct dyes for cellulosic textiles. Stud. Conserv..

[37] Türkmen N, Kirici S, Özgüven M, Inan M, Kaya DA (2004). An investigation of dye plants and their colourant substances in the eastern Mediterranean region of Turkey. Bot. J. Linnean Soc..

[38] Scarano P (2022). An endemic plant of the mediterranean area: phytochemical characterization of strawberry tree (*Arbutus unedo* L.) fruits extracts at different ripening stages. Front. Nutr..

[39] Scarano P (2020). Sustainability: Obtaining natural dyes from waste matrices using the prickly pear peels of *Opuntia ficus-indica* (L.) Miller. Agronomy.

[40] Lamia I, Zouhir C, Youcef A (2018). Characterization and transformation of the *Opuntia ficus indica* fruits. J. Food Measur. Characteriz..

[41] Sáenz C, Estévez AM, Sepúlveda E, Mecklenburg P (1998). Cactus pear fruit: A new source for a natural sweetener. Plant Foods Hum. Nutr..

[42] Scarano P (2022). Recovery and valorization of bioactive and functional compounds from the discarded of *Opuntia ficus-indica* (L.) Mill. Fruit Peel. Agron..

[43] Huang D, Boxin OU, Prior RL (2005). The chemistry behind antioxidant capacity assays. J. Agric. Food Chem..

[44] El-Sayed MA, Abbas FA, Refaat S, El-Shafae AM, Fikry E (2021). UPLC-ESI-MS/MS profile of the ethyl acetate fraction of aerial parts of bougainvillea ‘scarlett o’hara’ cultivated in egypt. Egypt J. Chem..

[45] Carini M, Aldini G, Furlanetto S, Stefani R, Facino RM (2001). LC coupled to ion-trap MS for the rapid screening and detection of polyphenol antioxidants from *Helichrysum stoechas*. J. Pharm. Biomed. Anal..

[46] Kiehne A, Engelhardt UH (1996). Thermospray-LC-MS analysis of various groups of polyphenols in tea: I. Catechins, flavonol O-glycosides and flavone C-glycosides. Zeitschrift fur Lebensmittel -Untersuchung und -Forschung.

[47] Määttä KR, Afaf Kamal-Eldin A, Törrönen R (2003). High-performance liquid chromatography (HPLC) analysis of phenolic compounds in berries with diode array and electrospray ionization mass spectrometric (MS) detection: Ribes Species. J. Agric. Food Chem..

[48] Sanjeeda I, Ansari TN (2014). Natural dyes: Their sources and ecofriendly. J. Environ. Res. Dev..

[49] Singh SV, Purohit MC (2012). Applications of eco-friendly natural dye on wool fibers using combination of natural and chemical mordants. Univ. J. Environ. Res. Technol..

[50] Zubairu A, Mshelia YM (2015). Effects of selected mordants on the application of natural dye from onion skin (*Allium cepa*). Sci. Technol..

[51] Khan AA (2014). Extraction of natural dye from red calico leaves: Gamma ray assisted improvements in colour strength and fastness properties. Dyes Pigments.

[52] Mikropoulou E, Tsatsaroni E, Varella EA (2009). Revival of traditional European dyeing techniques yellow and red colorants. J. Cult. Herit..

[53] Toussirot M (2013). Dyeing properties, coloring compounds and antioxidant activity of Hubera nitidissima (Dunal) Chaowasku (Annonaceae). Dyes Pigments.

[54] IUNC 2024. The IUCN Red List of Threatened Species - Rhus coriaria (Sumac). https://www.iucnredlist.org/species/63485/112727303 (2017).

[55] IUNC (1989). *IUNC SSC Position Statement on Research Involving Species at Risk of Extinction*. (1989).

[56] Dewanto V, Xianzhong W, Adom KK, Liu RH (2002). Thermal processing enhances the nutritional value of tomatoes by increasing total antioxidant activity. J. Agric. Food Chem..

[57] Tetko IV, Tanchuk VY, Kasheva TN, Villa AEP (2001). Estimation of aqueous solubility of chemical compounds using E-state indices. J. Chem. Inf. Comput. Sci..

[58] Chan-Bacab MJ (2015). Characterization and dyeing potential of colorant-bearing plants of the Mayan area in Yucatan Peninsula, Mexico. J. Clean Prod..

[59] Alessi, P. J. *et al. CIE—Internation Commission on Illumination Technical Report—Colorimetry*. (2004) 10.1364/JOSA.64.000210.

[60] Prieto B, Ferrer P, Sanmartín P, Cárdenes V, Silva B (2011). Color characterization of roofing slates from the Iberian Peninsula for restoration purposes. J. Cult. Herit..

[61] Sumner LW (2007). Proposed minimum reporting standards for chemical analysis: Chemical Analysis Working Group (CAWG) Metabolomics Standards Initiative (MSI). Metabolomics.

