# Supplementary material for: Rhus coriaria L. in tradition and innovation like natural dye
Source: Sci Rep. 2024 May 27;14:12068. doi: 10.1038/s41598-024-62528-8 (PMC11130214; doi:10.1038/s41598-024-62528-8)
Supplement: Supplementary file 1 — Supplementary Information. [file 41598_2024_62528_MOESM1_ESM.docx]

| **Supplementary Table 1.** pH, specific gravity and dry residue *R. coriaria* extracts. Values are presented as mean (triplicate) ± SD (standard deviation). Different superscripts letters indicate significant differences of parameters (pH, Brix, Residue, Extract) for each solvent (H_2_O, EtOH, EtOH:H_2_O (8:2)) between the four extraction methods (ME, NE and UAE) at *p* < 0.05 (Tukey pot-hoc). | | | | | | | | | | | | |
| --- | --- | --- | --- | --- | --- | --- | --- | --- | --- | --- | --- | --- |
| *Fruit* | | | | | | | | | | | | |
| **Solvent** | **ME** | | | | **NE** | | | | **UAE** | | | |
|  | **pH** | **Brix** | **Residue** | **Extract** | **pH** | **Brix** | **Residue** | **Extract** | **pH** | **Brix** | **Residue** | **Extract** |
|  |  | **[°Bx]** | **(g∙mL^−1^)** | **(g∙g^-1^ D.W.)** |  | **[°Bx]** | **(g∙mL^−1^)** | **(g∙g^-1^ D.W.)** |  | **[°Bx]** | **(g∙mL^−1^)** | **(g∙g^-1^ D.W.)** |
| H_2_O | 6.82 ± 0.03 ^a^ | 0.9 ± 0.2 ^a^ | 0.0235 ± 0.0093 ^a^ | 0.2337 ± 0.0022 ^a^ | 6.97 ± 0.02 ^a^ | 1.0 ± 0.2 ^a^ | 0.0321 ± 0.008 ^c^ | 0.3197 ± 0.015 ^b^ | 6.71 ± 0.02 ^a^ | 1.1 ± 0.1 ^a^ | 0.0306 ± 0.011 ^b^ | 0.3030 ± 0.0044 ^b^ |
| EtOH | 6.78 ± 0.02 ^a^ | 1.1 ± 0.1 ^a^ | 0.0199 ± 0.0054 ^a^ | 0.1944 ± 0.0032 ^a^ | 6.82 ± 0.07 ^a^ | 1.2 ± 0.1 ^a^ | 0.0203 ± 0.0072 ^a^ | 0.2007 ± 0.0023 ^a^ | 6.76 ± 0.03 ^a^ | 1.0 ± 0.2 ^a^ | 0.0180 ± 0.0075 ^a^ | 0.1773 ± 0.0014 ^a^ |
| EtOH:H_2_O  (8:2) | 6.79 ± 0.03 ^a^ | 1.0 ± 0.2 ^a^ | 0.0257 ± 0.006 ^a^ | 0.2549 ± 0.0039 ^a^ | 6.79 ± 0.04 ^a^ | 1.1 ± 0.2 ^a^ | 0.0269 ± 0.0042 ^a^ | 0.2661 ± 0.0021 ^b^ | 6.85 ± 0.02 ^a^ | 0.9 ± 0.1 ^a^ | 0.0253 ± 0.0051 ^a^ | 0.2524 ± 0.0031 ^a^ |
| *Leaves* | | | | | | | | | | | | |
| **Solvent** | **ME** | | | | **NE** | | | | **UAE** | | | |
|  | **pH** | **Brix** | **Residue** | **Extract** | **pH** | **Brix** | **Residue** | **Extract** | **pH** | **Brix** | **Residue** | **Extract** |
|  |  | **[°Bx]** | **(g∙mL^−1^)** | **(g∙g^-1^ D.W.)** |  | **[°Bx]** | **(g∙mL^−1^)** | **(g∙g^-1^ D.W.)** |  | **[°Bx]** | **(g∙mL^−1^)** | **(g∙g^-1^ D.W.)** |
| H_2_O | 6.89 ± 0.02 ^a^ | 0.8 ± 0.1 ^a^ | 0.0377 ± 0.0051 ^b^ | 0.3763 ± 0.0041 ^a^ | 6.79 ± 0.04 ^a^ | 0.8 ± 0.1 ^a^ | 0.0385 ± 0.0045 ^b^ | 0.3822 ± 0.0019 ^a^ | 6.80 ± 0.04 ^a^ | 1.0 ± 0.1 ^a^ | 0.0288 ± 0.0019 ^a^ | 0.2872 ± 0.0008 ^b^ |
| EtOH | 6.85 ± 0.02 ^a^ | 0.9 ± 0.1 ^a^ | 0.0087 ± 0.0002 ^a^ | 0.0868 ± 0.0021 ^a^ | 6.88 ± 0.03 ^a^ | 1.0 ± 0.2 ^a^ | 0.0106 ± 0.0022 ^a^ | 0.1030 ± 0.0012 ^b^ | 6.81 ± 0.03 ^a^ | 0.9 ± 0.1 ^a^ | 0.0017 ± 0.0003 ^a^ | 0.0168 ± 0.0017 ^a^ |
| EtOH:H_2_O  (8:2) | 6.86 ± 0.02 ^a^ | 0.9 ± 0.1 ^a^ | 0.0519 ± 0.0033 ^b^ | 0.5168 ± 0.0028 ^a^ | 6.84 ± 0.05 ^a^ | 1.0 ± 0.2 ^a^ | 0.0584 ± 0.0039 ^b^ | 0.5817 ± 0.0033 ^a^ | 6.85 ± 0.03 ^a^ | 0.9 ± 0.2 ^a^ | 0.0299 ± 0.0062 ^a^ | 0.2969 ± 0.0048 ^b^ |
| D.W. = dry weight, per extracted matrix | | | | | | | | | | | | |

| Supplementary Table 2. Total phenolic compound content (TPC) of *R. coriaria* extracts. Values are presented as mean (triplicate) ± SD (standard deviation). Different superscripts letters indicate significant differences of Total phenolic compound content for each solvent (H_2_O, EtOH, EtOH:H_2_O (8:2)) between the four extraction methods (ME, NE and UAE) at *p* < 0.05 (Tukey pot-hoc). | | | | | | | | | |
| --- | --- | --- | --- | --- | --- | --- | --- | --- | --- |
|  | **Total phenolic compound content (*C* GAE∙(mol·L^−1^))** | | | | | | | | |
|  | **ME** | | | **NE** | | | **UAE** | | |
|  | **H_2_O** | **EtOH** | **EtOH:H_2_O**  **(8:2)** | **H_2_O** | **EtOH** | **EtOH:H_2_O**  **(8:2)** | **H_2_O** | **EtOH** | **EtOH:H_2_O**  **(8:2)** |
| *Fruit* | 0.01060 ± 0.00287 ^a^ | 0.03055 ± 0.00151 ^a^ | 0.04223 ± 0.00655 ^a^ | 0.03675 ± 0.00814 ^b^ | 0.08148 ± 0.00249 ^b^ | 0.12490 ± 0.01746 ^c^ | 0.03926 ± 0.00450 ^b^ | 0.07583 ± 0.00660 ^b^ | 0.08602 ± 0.01957 ^b^ |
|  | | | | | | | | | |
| *Leaves* | 0.06447 ± 0.00497 ^a^ | 0.08135 ± 0.01541 ^a^ | 0.07010 ± 0.00469 ^a^ | 0.17132 ± 0.00163 ^b^ | 0.18108 ± 0.04112 ^b^ | 0.19297 ± 0.01525 ^b^ | 0.11622 ± 0.00804 ^ab^ | 0.13442 ± 0.01228 ^ab^ | 0.12359 ± 0.00478 ^ab^ |
| GAE expresses the Equivalents of Gallic Acid | | | | | | | | | |

| Supplementary Table 3. Colorimetric data. CIELAB colour coordinate values (CIELAB units) of discs cotton, linen and wool fibres coloured using the dye extract from *R. coriaria* fruits. Different superscripts letters indicate significant differences between treatments (U, DWM, DPL and DPA) at *p* < 0.05 (post-hoc Dunn) | | | | |
| --- | --- | --- | --- | --- |
| CIELAB Colour  Coordinates | U | DWM | DPL | DPA |
| Wool fibre | | | | |
| L* | 84.88 ± 2.20 ^a^ | 53.50 ± 7.50 ^b^ | 59.88 ± 4.88 ^b^ | 57.88 ± 6.60 ^b^ |
| a* | 1.25 ± 0.43 ^a^ | 31.75 ± 5.40 ^b^ | 28.00 ± 6.32 ^b^ | 29.25 ± 5.31 ^b^ |
| b* | 4.87 ± 0.93 ^a^ | 26.25 ± 2.28 ^b^ | 23.75 ± 2.77 ^b^ | 24.88 ± 2.37 ^b^ |
| C*_ab_ | 5.07 ± 0.84 ^a^ | 41.26 ± 5.39 ^b^ | 36.77 ± 6.60 ^b^ | 38.44 ± 5.50 ^b^ |
| h_ab_ | 74.93° ± 6.80° ^a^ | 39.93° ± 3.30° ^b^ | 40.84° ± 3.16° ^b^ | 40.74° ± 2.80° ^b^ |
| Linen fibre | | | | |
| L* | 91.50 ± 1.87 ^a^ | 74.36 ± 3.24 ^bc^ | 73.89 ± 2.79 ^b^ | 77.88 ± 3.67 ^ac^ |
| a* | −0.25 ± 0.08 ^a^ | 12.15 ± 1.65 ^ab^ | 13.53 ± 2.07 ^bc^ | 14.24 ± 1.83 ^c^ |
| b* | 9.00 ± 1.12 ^a^ | 24.08 ± 3.72 ^ac^ | 27.58 ± 4.91 ^b^ | 24.14 ± 3.81 ^bc^ |
| C*_ab_ | 9.03 ± 1.11 ^a^ | 26.97 ± 1.25 ^ac^ | 30.73 ± 2.03 ^b^ | 28.02 ± 2.54 ^bc^ |
| h_ab_ | 70.66° ± 5.33° ^ab^ | 63.23° ± 3.10° ^ab^ | 63.87° ± 4.86° ^a^ | 59.46° ± 1.29° ^b^ |
| Cotton fibre | | | | |
| L* | 87.75 ± 1.74 ^a^ | 48.00 ± 6.69 ^b^ | 56.25 ± 6.32 ^ab^ | 48.87 ± 6.90 ^b^ |
| a* | 1.38 ± 0.43 ^a^ | 43.00 ± 3.43 ^b^ | 37.12 ± 5.33 ^ab^ | 42.37 ± 7.30 ^b^ |
| b* | 2.62 ± 0.19 ^a^ | 31.25 ± 1.64 ^b^ | 32.75 ± 1.79 ^b^ | 33.75 ± 4.55 ^b^ |
| C*_ab_ | 3.00 ± 0.11 ^a^ | 53.17 ± 3.58 ^b^ | 49.57 ± 5.04 ^b^ | 54.20 ±8.44 ^b^ |
| h_ab_ | 61.53° ± 9.29° ^a^ | 36.07 ± 1.40° ^b^ | 41.69° ± 2.93° ^ac^ | 38.72° ± 1.76° ^bc^ |
| Data were expressed as the relative standard deviation, on measurements taken at random points on three replicates. U = Undyed. DWM = stained with extract dye without the use of mordants. DPL = pre-treatment with lemon juice. DPA = pre-treatment with aluminum potassium sulphate. | | | | |

| Supplementary Table 4. Colorimetric data. CIELAB colour coordinate values (CIELAB units) of discs cotton, linen and wool fibres coloured using the dye extract from *R. coriaria* leaves. Different superscripts letters indicate significant differences between treatments (U, DWM, DPL and DPA) at *p* < 0.05 (post-hoc Dunn) | | | | |
| --- | --- | --- | --- | --- |
| CIELAB Colour  Coordinates | U | DWM | DPL | DPA |
| Wool fibre | | | | |
| L* | 84.88 ± 2.20 ^a^ | 58.20 ± 5.20 ^b^ | 67.37 ± 4.39 ^ab^ | 57.13 ± 5.37 ^b^ |
| a* | 1.25 ± 0.43 ^a^ | -0.13 ± 1.17 ^ab^ | -1.38 ± 2.12 ^bc^ | -4.13 ± 1.17 ^c^ |
| b* | 4.87 ± 0.93 ^a^ | 19.88 ± 1.45 ^ab^ | 23.00 ± 1.00 ^bc^ | 32.63 ± 1.87 ^c^ |
| C*_ab_ | 5.07 ± 0.84 ^a^ | 19.91 ± 1.41 ^ab^ | 23.05 ± 1.01 ^bc^ | 32.91 ± 1.82 ^c^ |
| h_ab_ | 74.93° ± 6.80° ^ab^ | 18.97° ± 83.65° ^ac^ | -86.11° ± 1.16° ^b^ | -82.76° ± 2.16° ^bc^ |
| Linen fibre | | | | |
| L* | 91.50 ± 1.87 ^a^ | 85.34 ± 4.72 ^ab^ | 76.47 ± 5.61 ^c^ | 82.34 ± 2.84 ^bc^ |
| a* | −0.25 ± 0.08 ^a^ | 11.27 ± 2.56 ^ab^ | 16.42 ± 2.83 ^bc^ | 17.45 ± 4.24 ^c^ |
| b* | 9.00 ± 1.12 ^a^ | 30.05 ± 3.14 ^b^ | 27.54 ± 1.58 ^ac^ | 29.75 ± 3.28 ^bc^ |
| C*_ab_ | 9.03 ± 1.11 ^a^ | 32.09 ± 1.17 ^bc^ | 32.06 ± 1.90 ^ac^ | 34.49 ± 2.46 ^b^ |
| h_ab_ | 70.66° ± 5.33° ^ab^ | 69.44° ± 2.25° ^a^ | 59.20° ± 1.76° ^b^ | 59.61° ± 1.09° ^ab^ |
| Cotton fibre | | | | |
| L* | 87.75 ± 1.74 ^a^ | 54.25 ± 4.60 ^b^ | 69.25 ± 3.56 ^ac^ | 56.50 ± 6.30 ^bc^ |
| a* | 1.38 ± 0.43 ^a^ | 1.00 ± 0.71 ^ab^ | -1.50 ± 1.12 ^bd^ | -4.00 ± 1.41 ^cd^ |
| b* | 2.62 ± 0.19 ^a^ | 25.00 ± 1.32 ^ab^ | 25.13 ± 1.83 ^ab^ | 39.37 ± 2.59 ^b^ |
| C*_ab_ | 3.00 ± 0.11 ^a^ | 25.03 ± 1.34 ^ac^ | 25.20 ± 1.79 ^bc^ | 39.61 ± 2.48 ^b^ |
| h_ab_ | 61.53° ± 9.29° ^ab^ | -87.00° ± 0.99° ^a^ | -85.29° ± 2.08° ^c^ | -84.08° ± 2.30° ^bc^ |
| Data were expressed as the relative standard deviation, on measurements taken at random points on three replicates. U = Undyed. DWM = stained with extract dye without the use of mordants. DPL = pre-treatment with lemon juice. DPA = pre-treatment with aluminum potassium sulphate. | | | | |

| Supplementary Table 5. Wash and light fastness values for the colored fibres (*R. coriaria* fruits). | | | | |
| --- | --- | --- | --- | --- |
| Samples | Wash Fastness | Description | Light Fastness | Description |
| Wool |  |  |  |  |
| DWM(F) | 7 | Low loss of colouration | 7 | Acceptable loss of depth |
| DPL(F) | 8 | Acceptable loss of coloration | 8 | Slight e loss of depth |
| DPA(F) | 7-8 | Low loss of coloration | 8 | Slight loss of depth |
| Linen |  |  |  |  |
| DWM(F) | 5 | Visible loss of coloration | 5 | Visible loss of depth |
| DPL(F) | 5 | Visible loss of coloration | 6 | Low loss of depth |
| DPA(F) | 7-8 | Acceptable loss of coloration | 5 | Visible loss of depth |
| Cotton |  |  |  |  |
| DWM(F) | 7 | Low loss of coloration | 6-7 | Low loss of depth |
| DPL(F) | 8 | Slight loss of colouration | 9 | No loss of depth |
| DPA(F) | 7-8 | Low loss of colouration | 8 | Slight e loss of depth |
| F = fruits. U = Undyed. DWM = stained with extract dye without the use of mordants. DPL = pre-treatment with lemon juice. DPA = pre-treatment with aluminum potassium sulphate. | | | | |

| Supplementary Table 6. Wash and light fastness values for the colored fibres (*R. coriaria* leaves). | | | | |
| --- | --- | --- | --- | --- |
| Samples | Wash Fastness | Description | Light Fastness | Description |
| Wool |  |  |  |  |
| DWM(L) | 6 | Low loss of coloration | 6 | Low loss of depth |
| DPL(L) | 8 | Acceptable loss of coloration | 7 | Acceptable loss of depth |
| DPA(L) | 7 | Low loss of coloration | 7 | Acceptable loss of depth |
| Linen |  |  |  |  |
| DWM(L) | 5 | Visible loss of coloration | 5 | Visible loss of depth |
| DPL(L) | 6 | Visible loss of coloration | 6-7 | Low loss of depth |
| DPA(L) | 7–8 | Acceptable loss of coloration | 7-8 | Acceptable loss of depth |
| Cotton |  |  |  |  |
| DWM(L) | 6 | Low loss of coloration | 5-6 | Visible loss of depth |
| DPL(L) | 7–8 | Acceptable loss of coloration | 7-8 | Acceptable loss of depth |
| DPA(L) | 8–9 | Slight loss of coloration | 7-8 | Acceptable loss of depth |
| F = fruits. U = Undyed. DWM = stained with extract dye without the use of mordants. DPL = pre-treatment with lemon juice. DPA = pre-treatment with aluminum potassium sulphate. | | | | |

| Supplementary Table 7. Extraction instrument setting and method explication | | | |
| --- | --- | --- | --- |
| Techniques | **Instrument** | **Solvent** | **Method and General setting** |
| ME | - | H_2_O, EtOH, EtOH:H_2_O (8:2) | - Extraction time: 1 h; - Extraction temperature: 25.00 ± 1.00 °C; - Matrix quantity: 10 g (fruit and leaf matrix); - Solvent volume: 100 mL; - Method: the extraction was conducted under stirrer condition. |
| NE | Extractor Naviglio®  (ATLAS FILTRI ITALIA, Limena, Padua, Italy) | “ | - Extraction time: 1 h; - Extraction temperature: 25.00 ± 1.00 °C; - Matrix quantity: 10 g (fruit and leaf matrix); - Solvent volume: 100 mL; - Method: The extraction cycles consisted of a static phase (for a time of 2 min) and a dynamic phase (for a time of 2 min), which followed each other repeatedly: the total extraction time was, as in the other cases 1 h, comprising a total number of 15 cycles. |
| UAE | HEILSCHER UP200S  (Teltow, Germany) | “ | - Extraction time: 1 h; - Extraction temperature: 25.00 ± 1.00 °C; - Matrix quantity: 10 g (fruit and leaf matrix); - Solvent volume: 100 mL; - Wave power:24 kHz. |
|  | | | |


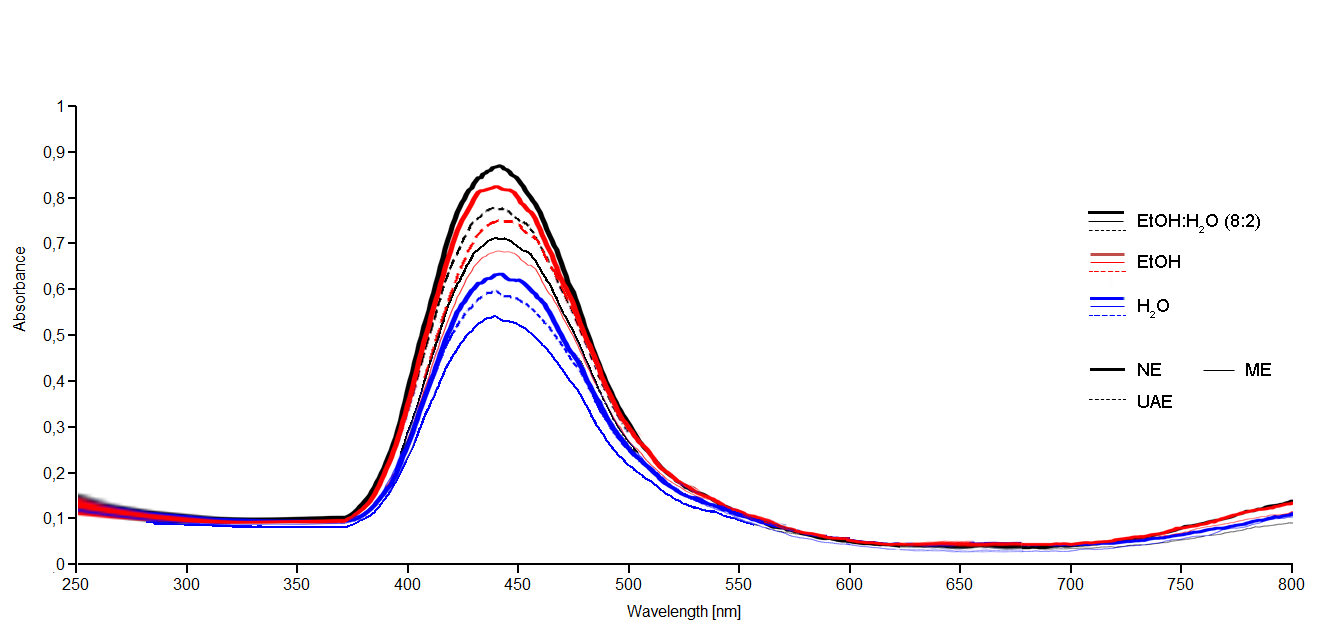


**Supplementary Figure 1.** UV-vis spectra of *R. coriaria* fruit extracts.


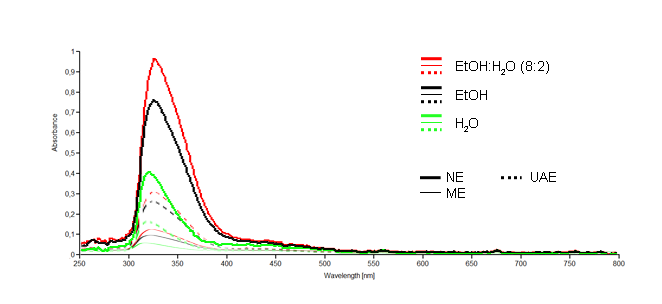


**Supplementary Figure 2.** UV-vis spectra of *R. coriaria* leaf extracts.

**
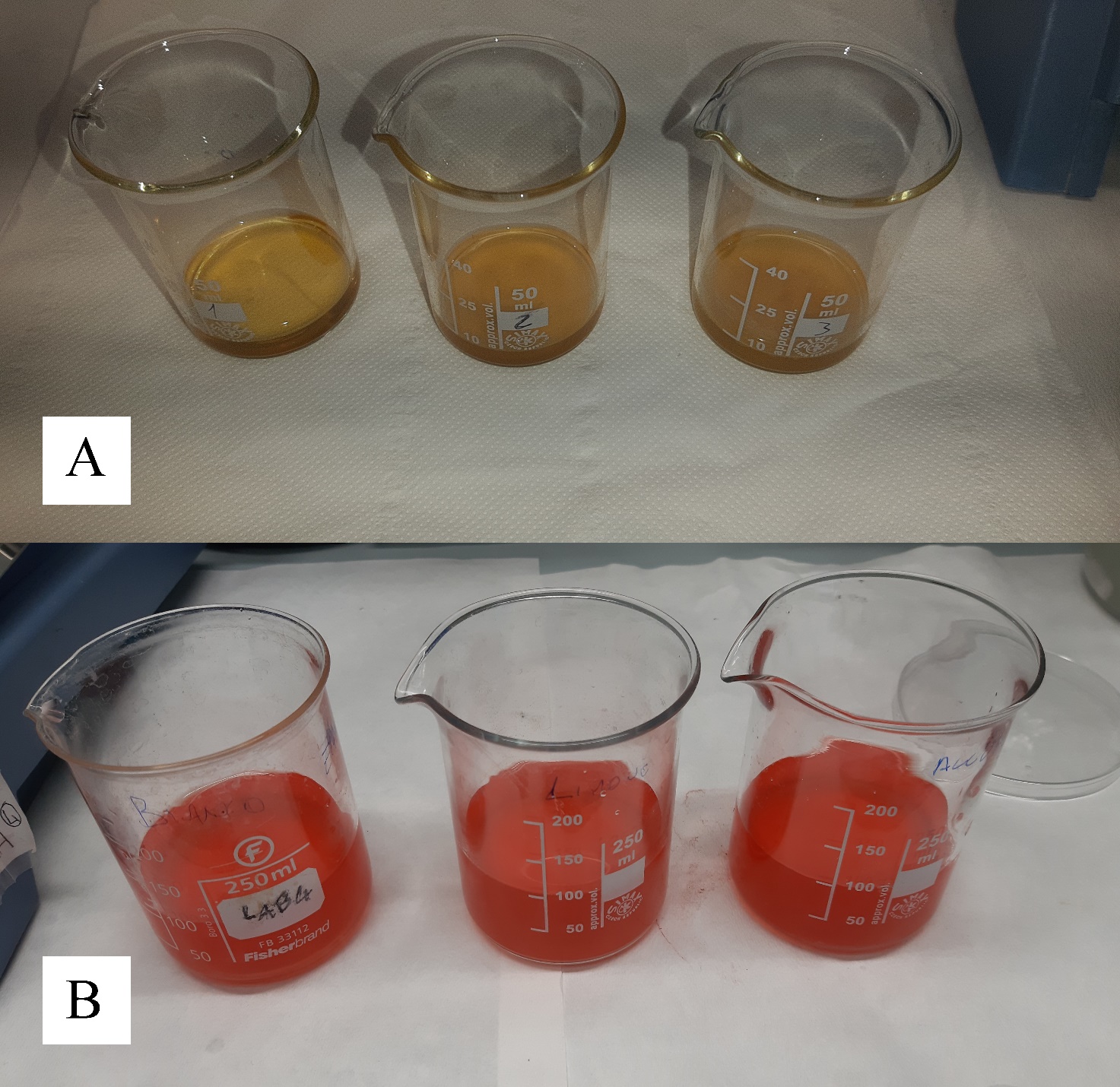
**

**Supplementary Figure 3.** Colorants extracted from leaf (A) and fruit (B): from left to right in solvents E8, E1 and H1.

**
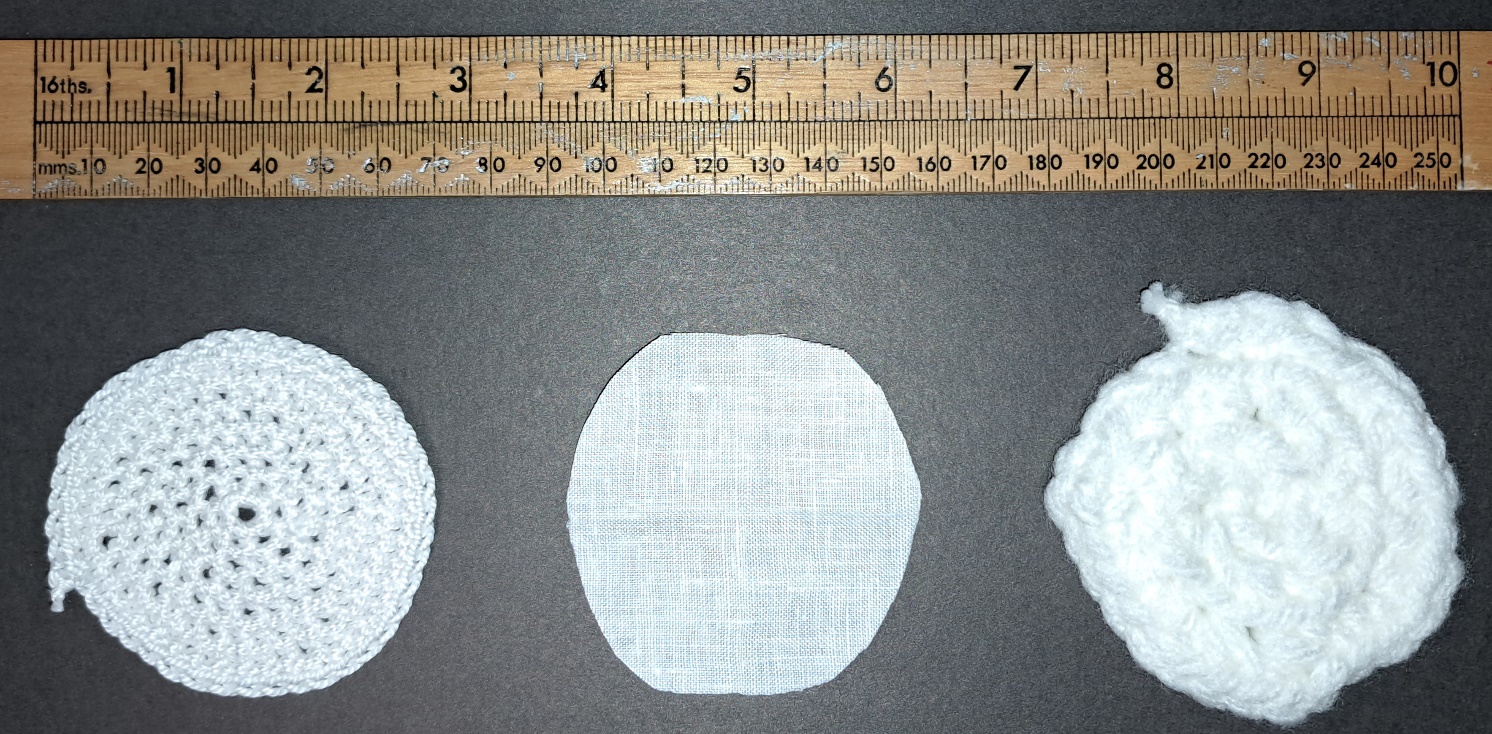
**

**Supplementary Figure 4.** Image of discs cotton (right), linen (middle) and wool (left) fibres before staining and dyeing treatments. These fibres are used as a reference for staining appearing as unstained fibres (U).


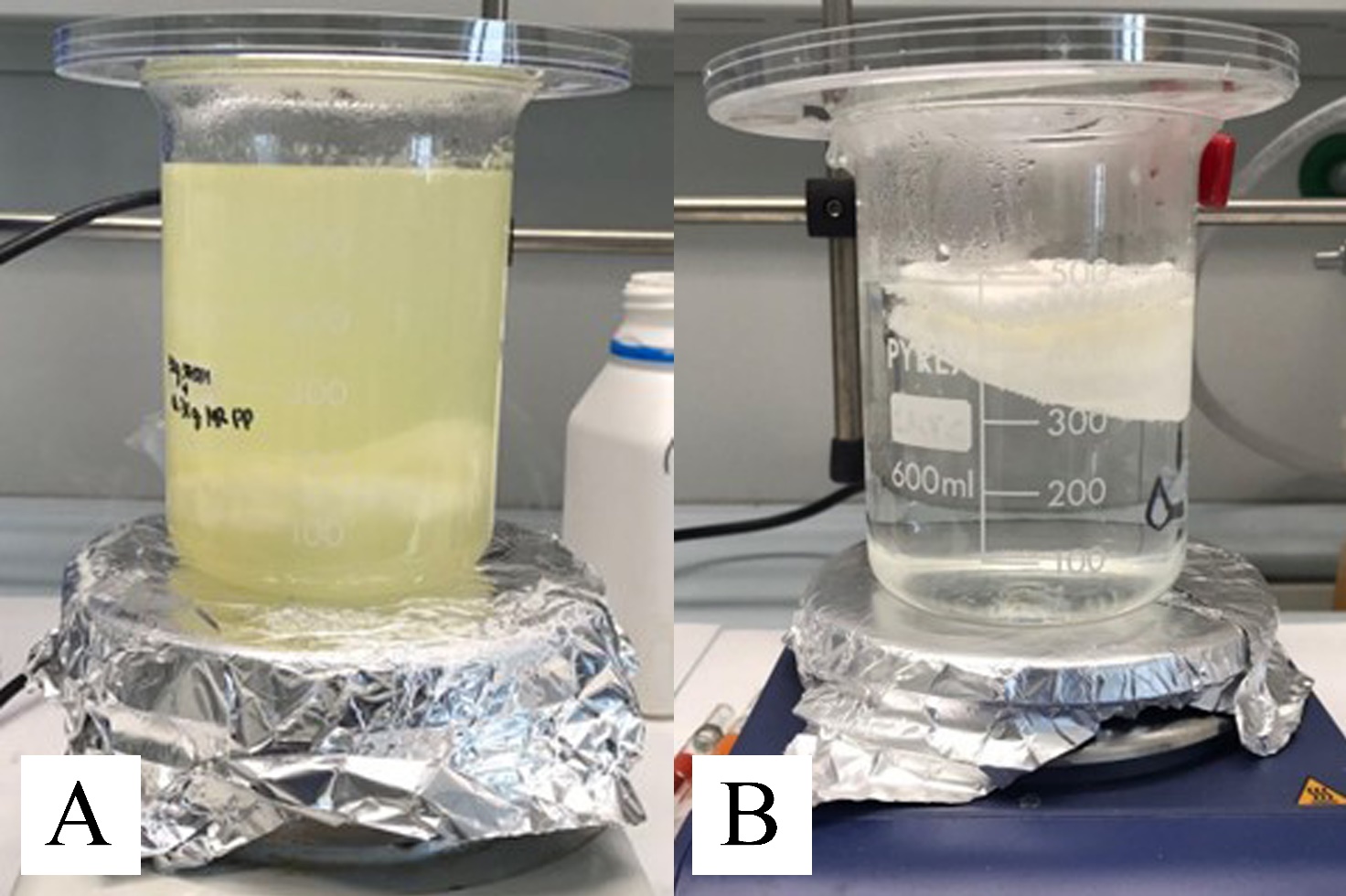


**Supplementary Figure 5.** Fiber mordant process: (A) lemon mordant and (B) rock alum mordant.


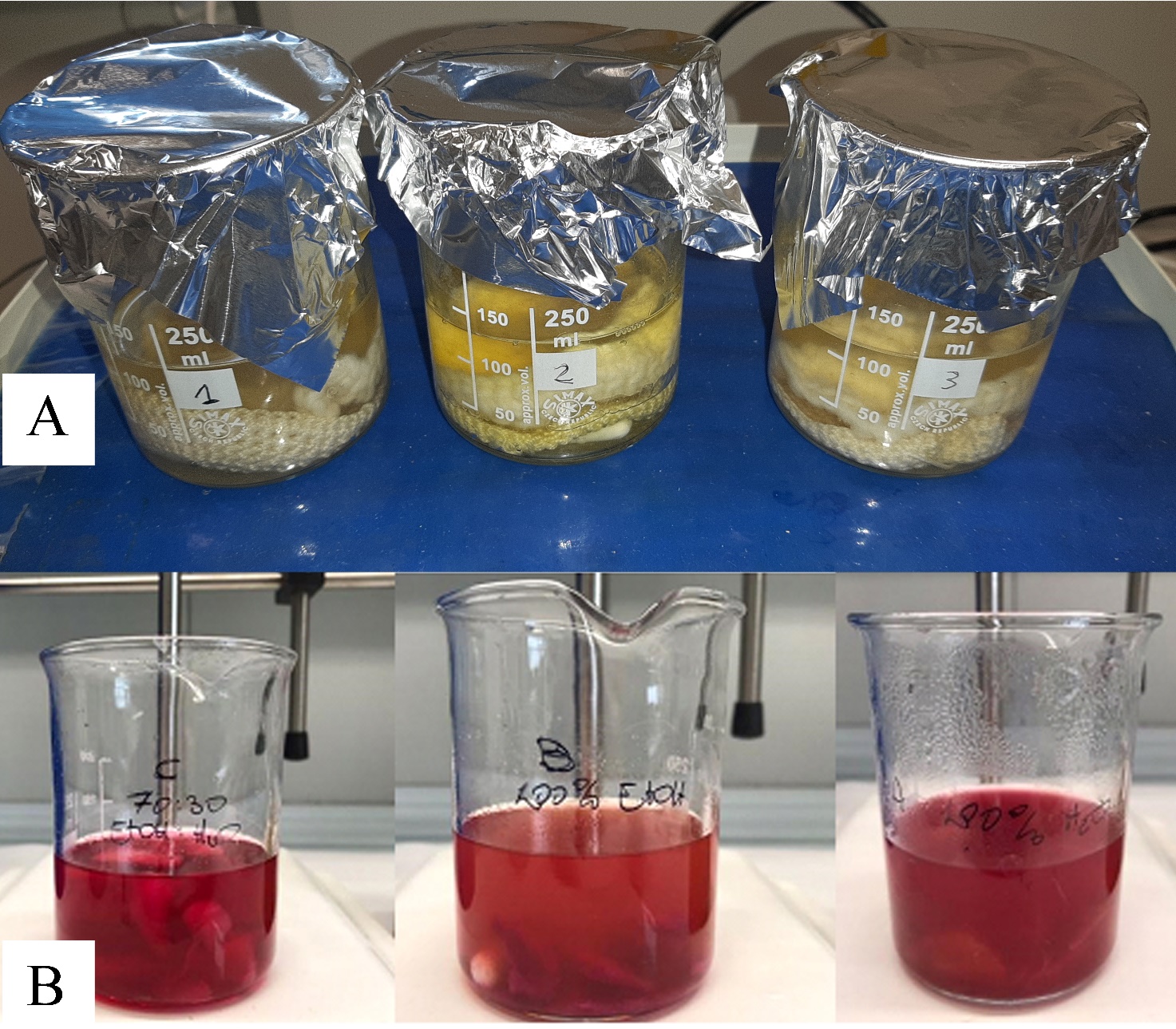


**Supplementary Figure 6.** Fibres process couloration whit different mordants. From left to right: DWM, DPL and DPA.
